# Chirality recognition of winding vine-shaped heterobiaryls with molecular asymmetry. Kinetic and dynamic kinetic resolution by Shi’s asymmetric epoxidation

**DOI:** 10.1038/s41598-018-19878-x

**Published:** 2018-01-26

**Authors:** Kazuki Maruhashi, Yoichi Okayama, Ryo Inoue, Shiomi Ashida, Yuka Toyomori, Kentaro Okano, Atsunori Mori

**Affiliations:** Department of Chemical Science and Engineering, Kobe University, 1-1 Rokkodai, Nada, Kobe 657-8501 Japan

## Abstract

The chirality of winding vine-shaped heterobiaryls with molecular asymmetry is recognized by a sugar-based chiral oxidant. Kinetic resolution of (±)-bisbenzoimidazole bearing an olefin moiety takes place with Shi’s asymmetric epoxidation to observe *k*_rel_ value up to ca. 35 affording the corresponding epoxide. The reaction of a (±)-bithiophene derivative also recognized the chirality to give the corresponding epoxide with er of 96:4 at 39% conversion. Dynamic kinetic resolution is found to take place when unsymmetrical biaryl composed of benzoimidazole/thiophene is subjected to Shi’s epoxidation, whose conversion of the racemic substrate exceeds to 50%.

## Introduction

Design of organic molecules with a novel class of molecular asymmetry is a challenging issue in the field of chemistry. A wide range of organic molecules showing molecular asymmetry have been shown to date and employed as chirality recognition and enantioselective catalysis, which bring about enantiospecific production of a variety of chiral molecules^1–5^. Such compounds also involve potential utilization as organic/polymeric advanced materials representative as chiral dopant of liquid crystals, circularly polarized luminescence, etc^6–9^. Binaphthyl derivatives with axial chiralities are shown to serve as a highly effective catalyst for asymmetric synthesis providing a wide range of chiral molecules with high enantiopurities^10,11^. Organic and organometallic compounds show unique planar chiralities and employed as catalysis and resolution^12–15^. Several helical molecules are employed as materials for the effective recognition of chiral small molecules^9,16–18^.

**Figure 1:**
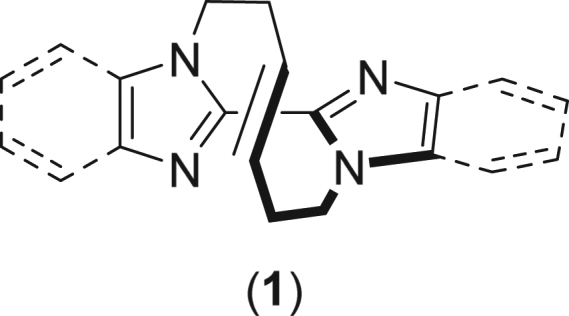
Structure of winding-vine-shaped heterobiaryl with molecular asymmetry.

We have recently designed and synthesized macrocyclic olefins incorporated in rigid heterobiaryls and such molecules thus obtained exhibit molecular asymmetry. In these molecules, axial, helical, and planar chiralities are induced along the heterobiaryl structure and we defined such a new class of chirality as *winding vine-shaped molecular asymmetry*^19–24^. (Fig. 1) Several compounds are revealed to be separated into each enantiomer via analytical/preparative chiral columns of HPLC and their chemical behaviors and spectroscopic properties are studied. Worthy of note is that the synchronized inversion of chiralities of axial, helical, and planar takes place to observe no epimer derived from such chiralities during isomerization, accordingly^23,24^. We have also shown that introduction of functional groups on the heteroaromatic ring allows metal-catalyzed coupling reactions^23^ as well as the double bond in the vine-shaped molecule allowed *cis*-addition reactions^20,23^. Thereby, these compounds allow potential application toward catalysis as an organic molecule^11,15^ by itself or as a ligand of metals^4,10,14^, recognition of external chiralities^5,9^, and formation of supramolecular compounds through self organization^6,7^.

Our concern turned to utilization of the formed compounds showing molecular asymmetry to application for the recognition of chiralities through the transformation reaction of functionalities of the vine-shaped molecule. We herein report our preliminary findings concerning that the winding-vine shaped molecular asymmetry successfully recognizes a sugar-derived molecule and the oxidation reaction at the olefin moiety lead to kinetic and dynamic kinetic resolutions^25^.

## Results and Discussion

**Figure 2:**
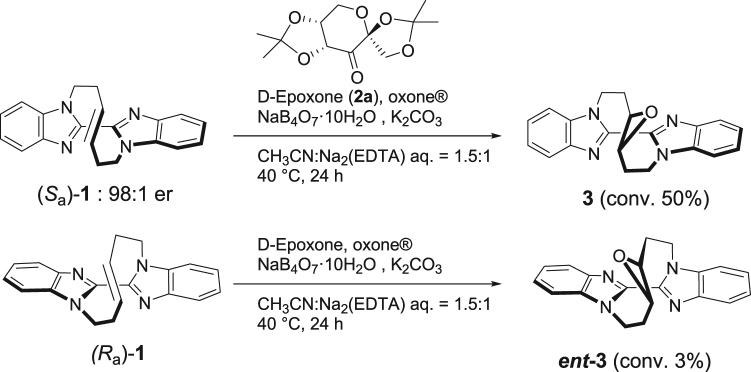
Scheme on rate difference of bisbenzoimidazole 1 of both enantiomers by Shi’s asymmetric epoxidation.

We first examined to treat bisbenzoimidazole **1** with a chiral fructose-derived dioxirane, which is formed by the reaction of D-epoxone (**2a**) and oxone^®^ (KHSO_5_·1/2KHSO_4_·1/2K_2_SO_4_), with which it was shown to afford the epoxide of a simple olefin developed by Shi and coworkers^26–28^. It was found that the chiral oxidant underwent enantiospecific epoxidation of **1**. When bisbenzoimidazole (+)-(*S*_a_)-**1** (>99% er) was subjected to the epoxidation reaction applying the standard conditions by Shi, oxidation of the olefin moiety of **1** proceeded smoothly and 50% of (+)-(*S*_a_)-**1** was converted to the corresponding epoxide **3**. The enantiopurity of **3** was confirmed as >99% er suggesting that no epimerization took place during the reaction^23^. On the other hand, the reaction of (−)-(*R*_a_)-**1** under similar conditions hardly proceeded (3%) to recover the unreacted (*R*_a_)-**1** (>99% er). (Scheme 1) These results show that chirality of the vine-shaped compound **1** recognize the chirality of sugar derivative **2a**. The matched combination of (+)-(*S*_a_)-**1** led to the epoxide **3** while mismatched (−)-(*R*_a_)-**1** remained unreacted as shown in Fig. 2.

**Figure 3:**
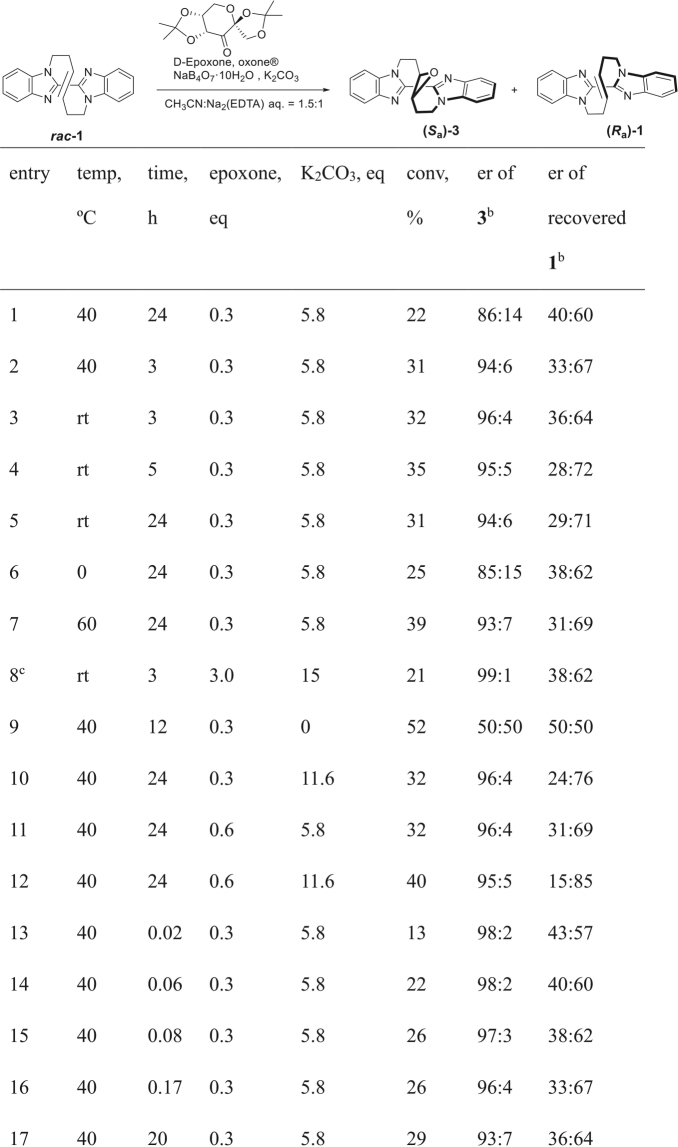
Table of the results on kinetic resolution of racemic bisbenzoimidzole **1** by Shi’s asymmetric epoxidation^a^. ^a^The reaction was carried out with 0.1 mmol of *rac***-**(±)**-1** with oxone^®^ (2.7 eq), ^*n*^Bu_4_NHSO_4_ (4 mol %), and NaB_4_O_7_ (0.5 eq) in CH_3_CN/aq. Na_2_(edta) (1.5:1 v/v). ^b^The enantiomeric ratio (er) was determined by HPLC analysis with chiral column (DAICEL Chiralpak IF). ^c^The reaction was carried out with NaHCO_3_ instead of K_2_CO_3_ in CH_3_CN/Na_2_(edta) aq.

As such successful chirality recognition of the winding vine-shaped compound was in hand by Shi’s epoxidation, the reaction was applied to the kinetic resolution of racemic (±)-**1** as summarized in the table of Fig. 3^29–32^. The reaction of **1** was carried out with 30 mol % of D-epoxone (**2a**) and oxone (2.7 eq) in the presence of 0.5 eq of NaB_4_O_7_·10H_2_O in a mixed solution of acetonitrile and aq. Na_2_(edta) (1.5:1) for 24 h. Epoxide **3** was obtained at 22% conversion with the enantiomeric ratio of 86:14 accompanied by recovery of unreacted **1** (40:60 er) (entry 1). Shorter reaction periods resulted in a lower conversion whereas the enantiomeric ratio of the obtained epoxide was slightly higher. (entry 2) The reaction at lower temperatures was also found to proceed, however, further improved selectivity was not achieved (entry 3, 4) and stirring a longer period suggested little improvement in the selectivity. (entry 5) The reaction at 0 °C as well as at higher temperature for 24 h did not improve the %conversion value and the enantiomeric ratio of **3**. (entry 6, 7). Switching the base to NaHCO_3_ (15 equiv.) instead of K_2_CO_3_ showed much higher selectivity but low conversion (21%). (entry 8) It was found important to perform the reaction in the presence of base, otherwise, no selectivity was observed at all(According to Shi’s discussion on the asymmetric epoxidation, undesired decomposition of D-epoxone by Baeyer-Villiger oxidation takes place without K_2_CO_3_, which inhibits the progress of epoxidation. See: ref.^26^). (entry 9) Indeed, increased amount of K_2_CO_3_ resulted in giving improved enantiomeric ratio of **3** (entry 10 vs. 1). Use of increased amount of D-epoxone was also effective to result in a high enantiomeric ratio at 32% conversion (entry 11). The highest conversion with formation of a high degree of enantioselectivity of epoxide **3** was achieved when 0.6 eq of D-epoxone (**2a**) and 11.6 eq of K_2_CO_3_ (95:5 er at 40% conv.: entry 12), accordingly. It was found that longer reaction period resulted in slight decrease of the enantiomeric ratio of **3**. Non-enantioselective epoxidation by acetone, which was formed by decomposition of D-epoxone, would occur along with the prolonged reaction periods. In the early stage of the reaction, the enantiomeric ratio of the obtained epoxide **3** was much higher. (entry 13–17) Employing the results on the reaction period of <5 min, the relative rate of the formation of **3** was estimated as *k*_rel_ = 35.0.

**Figure 4:**
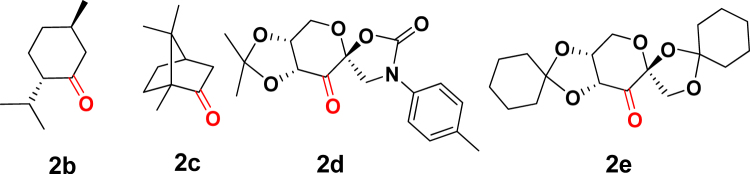
Chiral ketones for the precursor of dioxirane.

**Figure 5:**
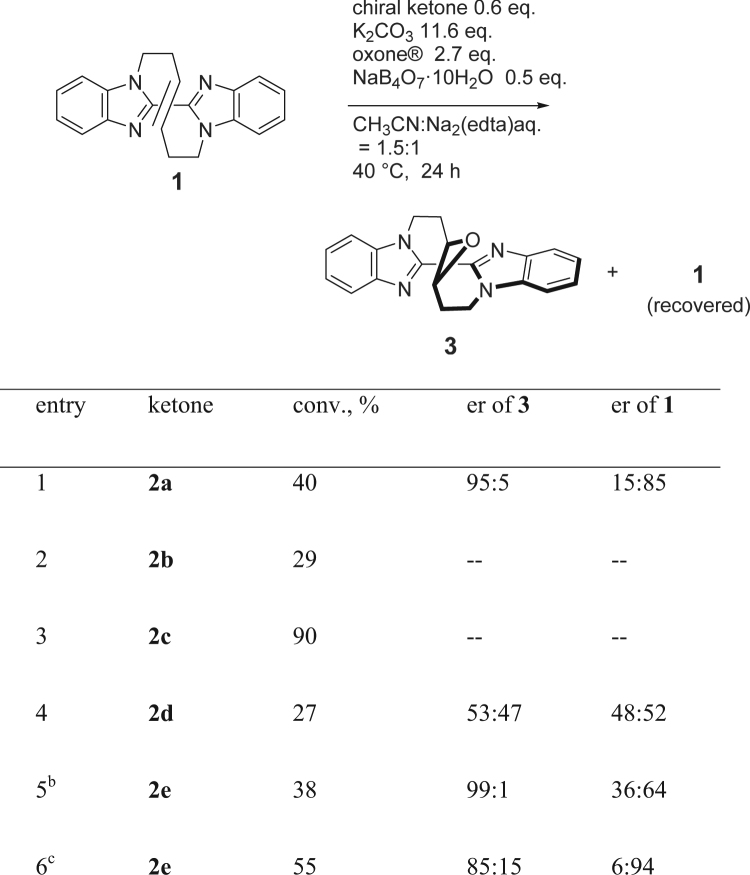
Table of the results on kinetic resolution of the vine-shaped bisbenzoimidazole **1** by asymmetric epoxidation with several chiral ketones^a^. ^a^Unless noted, the reaction was carried out with 0.6 eq of chiral ketone, oxone^®^ (2.7 eq), and K_2_CO_3_ (11.6 eq) in CH_3_CN/aq. ^b^The reaction was carried out at 60 °C for 24 h. ^c^The reaction period: 48 h.

We then studied the use of chiral ketone in the kinetic resolution by asymmetric epoxidation. Figure 4 summarizes the employed ketones for the reaction. In addition to natural terpene (−)-menthone (**2b**) and (+)-camphor (**2c**), chiral ketone **2d** was prepared from D-glucose by following the literature procedure^33^. A fructose-derived chiral ketone bearing a different protective group was also prepared using cyclohexanone instead of acetone leading to cyclohexylidene derivative **2e**^34^. As summarized in Fig. 5, use of **2b** as a chiral ketone resulted in showing little rate difference between enantiomers at 29% conversion. (entry 2) Although the reaction with **2c** as a chiral ketone proceeded smoothly to afford 90% of epoxide suggesting that both enantiomers were converted into epoxide. (entry 3) The glucose derived **2d** was found to be converted into epoxide in 27% yield, however, little enantiodifferentiation was observed in the reaction. (entry 4) A remarkable selectivity was observed in the use of cylohexylidene derivative **2e**. Epoxide **3** of extremely high enantiomeric ratio (99:1) was obtained at the 38% conversion. (entry 5) On the other hand, the reaction at an improved conversion (55%) afforded highest enantiomeric ratio (6:94 er) of recovered bisbenzoimidazole **1** accompanied by 85:15 er of epoxide **3**. These results are summarized in Fig. 5.

**Figure 6:**
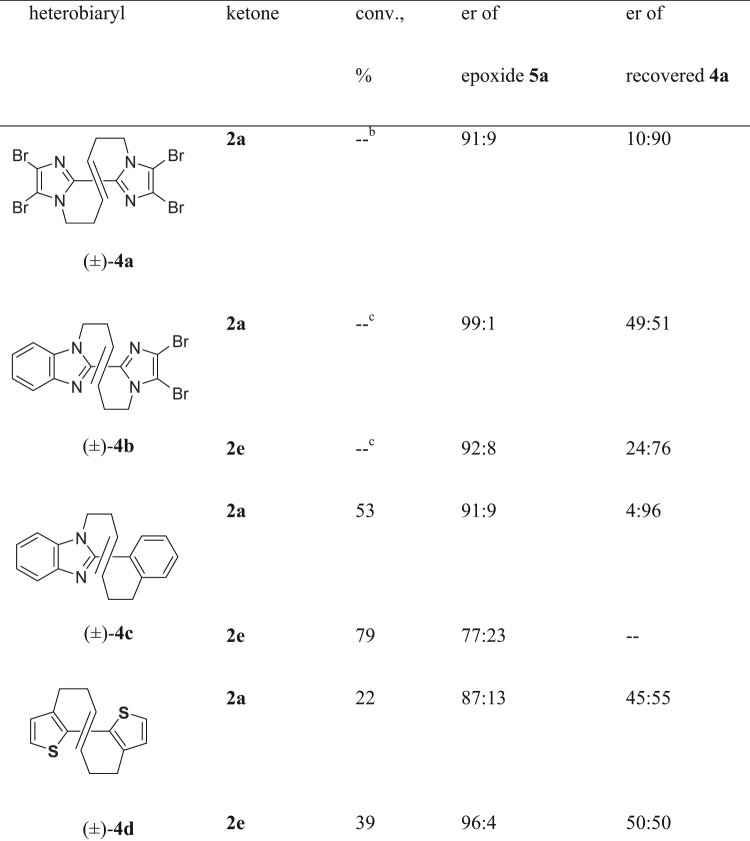
Table of the results on kinetic resolution of vine-shaped bithiophene **4** with chiral ketone **2a** and **2e**^a^. ^a^The reaction was carried out 0.6 eq of chiral ketone **2**, oxone^®^ (2.7 eq), ^*n*^Bu_4_NHSO_4_ (4.0 mol %), and K_2_CO_3_ (11.6 eq) in CH_3_CN/aq. Na_2_(edta) (1.5:1 v/v) at 40 °C for 24 h. ^b^The reaction proceeded to give epoxide **5a** accompanied by inseparable and unidentified by-products. The ratio of **4a**:**5a** was 50:50 by HPLC analysis. ^c^The ratio of **4b**:**5b** was 99:1 (with **2a**) and 62:38 (with **2e**), respectively, by HPLC analysis.

Kinetic resolution was then subjected to several vine-shaped heterobiaryls (±)-**4** in a similar manner as shown in Fig. 6. The reaction of tetrabromobisimidazole (±)-**4a** proceeded to afford epoxidation product **5a** in a similar selectivity accompanied by formation of inseparable and unidentified by-products. Use of unsymmetrical heterobiaryl composed of benzoimidazole and dibromoimidazole (±)-**4b**^22^ showed the similar selectivity to give **5b**, however, the reaction also resulted to afford unidentified by-products. Kinetic resolution of heterobiaryl composed of benzoimidazole and benzene (±)-**4c**^22^ was found to proceed smoothly to afford epoxide **5c** at 53% conversion with er of 91:9 along with the recovered **4c** (er = 4:96). It was also found that bithiophene (±)-**4d** underwent enantiospecific oxidation with D-epoxone (**2a**) leading to the corresponding epoxide **5d** (87:13 er at 22% conversion). Slightly improved selectivity and conversion were achieved when oxidant bearing the cyclohexylidene moiety **2e** was employed. The obtained epoxide (±)-**5d** exhibited 96:4 er at 39% conversion. Despite enantioselective consumption of (±)-**4d** to give **5d** with a high enantiomeric ratio worthy of note is that the recovered bithiophene **4d** was mostly racemic (45:55–50:50)(Racemization barrier was estimated by bithiophene **4d** was experimentally estimated as 101.69 kJ mol^−1^ and calculated as 106.15 kJ mol^−1^, while that of bisbenzoimidazole **1** was 130.15 kJ mol^−1^ (experimental) and 138.59 kJ mol^−1^ (calculated). Results of other compounds: See Supporting Information). These results suggest that isomerization of recovered **4d** to the racemate took place during enantioselective epoxidation reaction implying possible dynamic kinetic resolution^35–38^ whereas conversion of the reaction did not exceed 50%.

**Figure 7:**
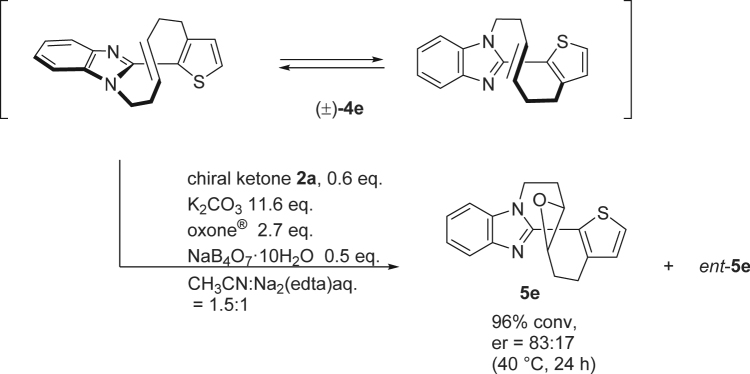
Scheme on the dynamic kinetic resolution of winding-vine shaped heterobiaryl **4e** with Shi’s oxidation.

Accordingly, we surveyed structures of the winding-vine shaped biaryl that undergo racemization in a comparable rate to the Shi’s oxidation under mild conditions. It was found that unsymmetrical heterbiaryl composed of benzoimidazole and another aromatic ring^22^. As depicted in Fig. 7, racemic heterobiaryl composed of benzoimidazole and thiophene (±)-**4e** was subjected to the epoxidation reaction with D-epoxone (**2a**). The reaction was found to proceed at 40 °C at 96% conversion after stirring for 24 h. The enantiomeric ratio of the obtained epoxide **5e** was confirmed to be 83:17, which value exceeded the theoretical value in the case of non-dynamic resolution (52:48). The result suggests that racemization of **4e** accompanies the Shi’s epoxidation and thus showed that dynamic kinetic resolution of (±)-**4e** indeed took place.

In summary, we have shown that winding vine-shaped heterobiaryl with molecular asymmetry bearing an olefin moiety recognizes chirality of sugar-derived chiral ketone **2a** and **2e** to induce the rate difference between enantiomers of heterobiaryl. Kinetic resolution was thus achieved when a racemic heterobiaryl derivative was subjected to the conditions of Shi’s asymmetric epoxidation. Heterobiaryl (±)-**4** composed of imidazoles and thiophenes showed remarkable rate difference between enantiomers. It was also found that unsymmetrical biaryl bearing benzoimidazole/thiophene (±)-**4e** indicated dynamic kinetic resolution at the conversion to the epoxide exceeding to 50%.

## Materials and Methods

All the reactions were carried out under nitrogen atmosphere. ^1^H NMR (300, 400 MHz) and ^13^C NMR (100, 125 MHz) spectra were measured on JEOL ECZ400, Varian Gemini 300, or Bruker Avance 500 spectrometer. Unless noted, NMR spectra were measured at room temperature. The chemical shift was expressed in ppm with CHCl_3_ (7.26 ppm for ^1^H), CDCl_3_ (77.0 ppm for ^13^C) as internal standards. High resolution mass spectra (HRMS) were measured by JEOL JMS-T100LP AccuTOF LC-Plus (ESI) with a JEOL MS-5414DART attachment. For thin layer chromatography (TLC) analyses throughout this work, Merck precoated TLC plates (silica gel 60 F_254_) were used. Purification by HPLC with preparative SEC column (JAI-GEL-2H) was performed by JAI LC-9201. HPLC by chiral column was performed with JASCO LC-2000 Plus using DAICEL Chiralpak IC or IF (0.46 mm id, 25 cm length) with the flow rate = 1.0 mL/min unless noted. Chemicals were purchased and used without further purification unless noted. Bisbenzoimidazole **1** was prepared according to the procedure described in our previous report^19^. Separation of racemic **1** by preparative HPLC with chiral column was carried out with DAICEL Chiralpak IF (20 mm id, 25 cm length). L-Menthone and D-Camphor were purchased and used without further purification. D-epoxone was purchased from Alfa-Aesar Co. Ltd. Other chiral ketones for Shi’s asymmetric epoxidation were prepared according to the reported procedures^33,34^. Tetrabromobisimidazole (±)-**4a** was prepared by the procedure in our previous report^23^. Unsymmetrical heterbiaryls (±)-**4b**, (±)-**4c**, and (±)-**4e** were prepared by the procedures in our previous report^22^. Bithiophene (±)-**4d** was prepared by the procedure described in our previous report^21^. Racemic epoxides **3** and **5d** were prepared by epoxidation of (±)-**1** and (±)-**4d** with *m*-chloroperbenzoic acid or oxone/acetone in a manner described previously^23^.

### Shi’s asymmetric epoxidation of enantiopure bisbenzoimidazole (*S*_a_)-(+)-1

In a screw capped test tube were placed a buffer solution composed of 0.05 M Na_2_B_4_O_7_·10H_2_O in 4 × 10^−4^ M aqueous Na_2_(EDTA) (0.7 mL), acetonitrile (1.5 mL), bisbenzoimidazole (*S*_a_)-**1** (31.4 mg, 0.1 mmol), D-epoxone (0.06 mmol), and tetrabutylammonium hydrogen sulfate (1.5 mg, 0.004 mmol). The reaction mixture was warmed to 40 °C and a solution of Oxone (166 mg, 0.27 mmol) in aqueous Na_2_(EDTA) (4 × 10^−4^ M, 1.0 mL) and aqueous solution of K_2_CO_3_ (160 mg, 1.16 mmol in 1.0 mL of water) were added successively in three portions with each 1 h interval. The reaction mixture was stirred at 40 °C for an additional 24 h. The reaction mixture was diluted with water and extracted twice with ethyl acetate. The combined organic layer was washed with brine, dried over anhydrous sodium acetate, and concentrated under reduced pressure to leave a crude oil. The enantioselectivity and conversion of the reaction was estimated by HPLC analysis with a chiral column (DAICEL Chiralpak IF) using hexane/ethanol = 1:1 as an eluent to show 50% conversion to **3** whose enantiomeric ratio was revealed as >99:1 (*t*_R_ = 15.0 min).

### Shi’s asymmetric epoxidation of enantiopure bisbenzoimidazole (*R*_a_)-(-)-1

The reaction was carried out in a similar manner under similar conditions as described above to result in 3% conversion by HPLC analysis with chiral column (DAICEL Chiralpak IF) to confirm recovery of unreacted (*R*_a_)-1 with e. r. of >99:1 (*t*_R_ = 8.8 min) using hexane/ethanol = 1:1 as an eluent.

### Kinetic resolution of racemic bisbenzoimidazole (±)-1 by Shi’s asymmetric epoxidation

The reaction was carried out in a similar manner to that of (*S*_a_)-(+)-**1** to show HPLC profile of *t*_R_ = 6.4 min ((*S*_a_)-**1**), 8.6 min ((*R*_a_)-**1**), 11.5 min (**3**), and 13.9 min (**3**), respectively, with a chiral column (DAICEL Chiralpak IF) using hexane/ethanol = 1:1 as an eluent.

### Kinetic resolution of racemic (±)-4d by Shi’s epoxidation

The reaction was carried out in a similar manner for the resolution of (±)-**1**. When the reaction was carried out with chiral ketone **2e** to undergo the reaction at 39% conversion. Epoxide **5d** was obtained with er of 96:4, which was confirmed by HPLC analysis with chiral column. (DAICEL Chiralpak IF, eluent: hexane/ethanol = 100:1, *t*_R =_ 10.9 and 11.9 min, flow rate = 1.0 mL/min) with recovery of **4d** as a mostly racemic mixture (ca. 50:50, *t*_R_ = 12.0 min and 13.4 min, eluent: hexane, flow rate = 0.5 mL/min).

Kinetic resolution of (±)-**4a**, (±)-**4b**, and (±)-**4c** was carried out in the above manner.

(±)-**4a**: *t*_R_ = 6.3 min, *t*_R_ = 10.4 min; **5a**: *t*_R_ = 8.6 min, *t*_R_ = 12.8 min, respectively, with chiral column. (DAICEL Chiralpak IF, eluent: hexane/ethanol = 1:1, flow rate = 1.0 mL/min. (±)-**4b**: *t*_R_ = 6.8 min, *t*_R_ = 8.8 min; **5a**: *t*_R_ = 11.6 min, *t*_R_ = 13.0 min, respectively, with chiral column. (DAICEL Chiralpak IF, eluent: hexane/ethanol = 1:1, flow rate = 1.0 mL/min. (±)-**4c**: *t*_R_ = 4.5 min; **5c**: *t*_R_ = 6.0 min, *t*_R_ = 6.6 min, respectively, with chiral column. (DAICEL Chiralpak IF, eluent: hexane/ethanol = 1:1, flow rate = 1.0 mL/min and *t*_R_ = 90 min and 93 min, respectively, with DAICEL Chiralpak IF, eluent: hexane/ethanol = 50:1, flow rate = 0.5 mL/min to show the ratio of ca. 96:4 (by curve fitting).

### Dynamic Kinetic resolution of racemic bithiophene ( ± )-4e by Shi’s epoxidation

The reaction was carried out in a similar manner as described above. When chiral ketone **2e** was employed for the reaction, HPLC analysis with chiral column (DAICEL Chiralpak IF, flow rate = 0.5 mL/min) using hexane/ethanol = 10:1 as an eluent for unreacted **4e** revealed to exhibit 4% recovery (*t*_R_ = 24.0 min, 29.3 min: 50:50 er) and epoxide **5e** with the enantiomeric ratio of 83:17 (*t*_R_ = 65.2 min, 72.1 min: 83:17 er).

## Electronic supplementary material

Supplementary Information

## References

[1] Eliel, E. L., Wilen, S. H. & Doyle, M. P. *Basic Organic Stereochemistry*. (Wiley, 2001).

[2] Adams R, Yuan HC (1933). The Stereochemistry of Diphenyls and Analogous Compounds. Chem. Rev..

[3] Marshall JA (1980). Trans-cycloalkenes and [a.b]betweenanenes, molecular jump ropes and double bond sandwiches. Acc. Chem. Res..

[4] Noyori R (1990). Chiral Metal Complexes as Discriminating Molecular Catalysts. Science.

[5] Feringa BL, van Delden RA, Koumura N, Geertsema EM (2000). Chiroptical Molecular Switches. Chem. Rev..

[6] Goodby JW (1991). Chirality in liquid crystals. J. Mater. Chem..

[7] Whitesides GM, Grzybowski B (2002). Self-Assembly at All Scales. Science.

[8] Sánchez-Carnerero EM (2015). Circularly Polarized Luminescence from Simple OrganicMolecules. Chem. Eur. J..

[9] Yashima E (2016). Supramolecular Helical Systems: Helical Assemblies of Small Molecules, Foldamers, and Polymers with Chiral Amplification and Their Functions. Chem. Rev..

[10] Noyori R (2002). Asymmetric Catalysis: Science and Opportunities (Nobel Lecture) Copyright© The Nobel Foundation 2002. We thank the Nobel Foundation, Stockholm, for permission to print this lecture. Angew. Chem. Int. Ed..

[11] Maruoka K, Ooi T (2003). Enantioselective Amino Acid Synthesis by Chiral Phase-Transfer Catalysis. Chem. Rev..

[12] Cope AC, Ganellin CR, Johnson HW, Van Auken TV, Winkler HJS (1963). Molecular Asymmetry of Olefins. I. Resolution of trans -Cyclooctene 1–3. J. Am. Chem. Soc..

[13] Urbano A (2003). Recent Developments in the Synthesis of Helicene-Like Molecules. Angew. Chem. Int. Ed..

[14] Dai L-X, Tu T, You S-L, Deng W-P, Hou X-L (2003). Asymmetric Catalysis with Chiral Ferrocene Ligands. Acc. Chem. Res..

[15] Fu GC (2004). Asymmetric Catalysis with ‘Planar-Chiral’ Derivatives of 4-(Dimethylamino)pyridine. Acc. Chem. Res..

[16] Suginome M, Yamamoto T, Nagata Y, Yamada T, Akai Y (2012). Catalytic asymmetric synthesis using chirality-switchable helical polymer as a chiral ligand. Pure Appl. Chem..

[17] Martin RH (1974). The Helicenes. Angew. Chemie Int. Ed. English.

[18] Buchmeiser, M. R. *Polymeric materials in organic synthesis and catalysis*. (Wiley, 2006).

[19] Nishio S (2012). Axially Chiral Macrocyclic E -Alkene Bearing Bisazole Component Formed by Sequential C–H Homocoupling and Ring-Closing Metathesis. Org. Lett..

[20] Okayama Y (2015). Enantioselective Synthesis of Macrocyclic Heterobiaryl Derivatives of Molecular Asymmetry by Molybdenum-Catalyzed Asymmetric Ring-Closing Metathesis. Angew. Chem. Int. Ed..

[21] Toyomori Y (2016). Bithiophene with Winding Vine-shaped Molecular Asymmetry. Preparation, Structural Characterization, and Enantioselective Synthesis. Bull. Chem. Soc. Jpn..

[22] Mori A (2017). Synthesis of Unsymmetrical Heterobiaryls with Winding Vine-Shaped Molecular Asymmetry through a Condensation Pathway. Heterocycles.

[23] Okayama Y, Maruhashi K, Tsuji S, Mori A (2015). Studies on Diastereoselective Functionalization, Optical Resolution, and Racemization Behaviors of Macrocyclic Bisimidazole of Winding-Vine-Shaped Molecular Asymmetry. Bull. Chem. Soc. Jpn..

[24] See also: Yoshioka S, Inokuma Y, Hoshino M, Sato T, Fujita M (2015). Absolute structure determination of compounds with axial and planar chirality using the crystalline sponge method. Chem. Sci..

[25] Kinetic resolution of planar chiral cyclic ether. See: Tomooka, K., Komine, N., Fujiki, D., Nakai, T. & Yanagitsuru, S. Planar Chiral Cyclic Ether: Asymmetric Resolution and Chirality Transformation. *J. Am. Chem. Soc*. **127**, 12182–12183 (2005). 10.1021/ja053347g 16131170

[26] Wang Z-X, Tu Y, Frohn M, Zhang J-R, Shi Y (1997). An Efficient Catalytic Asymmetric Epoxidation Method. J. Am. Chem. Soc..

[27] Shi Y (2004). Organocatalytic Asymmetric Epoxidation of Olefins by Chiral Ketones. Acc. Chem. Res..

[28] Zhu Y, Wang Q, Cornwall RG, Shi Y (2014). Organocatalytic Asymmetric Epoxidation and Aziridination of Olefins and Their Synthetic Applications. Chem. Rev..

[29] Katsuki T, Sharpless KB (1980). The first practical method for asymmetric epoxidation. J. Am. Chem. Soc..

[30] Martin VS (1981). Kinetic resolution of racemic allylic alcohols by enantioselective epoxidation. A route to substances of absolute enantiomeric purity?. J. Am. Chem. Soc..

[31] Larrow JF, Schaus SE, Jacobsen EN (1996). Kinetic Resolution of Terminal Epoxides via Highly Regioselective and Enantioselective Ring Opening with TMSN_3_. An Efficient, Catalytic Route to 1,2-Amino Alcohols. J. Am. Chem. Soc..

[32] Vedejs E, Jure M (2005). Efficiency in Nonenzymatic Kinetic Resolution. Angew. Chem. Int. Ed..

[33] Goeddel D (2006). Effective Asymmetric Epoxidation of Styrenes by Chiral Dioxirane. J. Org. Chem..

[34] Tu Y (1998). Structural Probing of Ketone Catalysts for Asymmetric Epoxidation. J. Org. Chem..

[35] Huerta FF, Minidis ABE, Bäckvall J-E (2001). Racemisation in asymmetric synthesis. Dynamic kinetic resolution and related processes in enzyme and metal catalysis. Chem. Soc. Rev..

[36] Pellissier H (2011). Recent developments in dynamic kinetic resolution. Tetrahedron.

[37] Bhat V, Welin ER, Guo X, Stoltz BM (2017). Advances in Stereoconvergent Catalysis from 2005 to 2015: Transition-Metal-Mediated Stereoablative Reactions, Dynamic Kinetic Resolutions, and Dynamic Kinetic Asymmetric Transformations. Chem. Rev..

[38] Noyori R, Tokunaga M, Kitamura M (1995). Stereoselective Organic Synthesis via Dynamic Kinetic Resolution. Bull. Chem. Soc. Jpn..

