# Supplementary material for: Chirality recognition of winding vine-shaped heterobiaryls with molecular asymmetry. Kinetic and dynamic kinetic resolution by Shi’s asymmetric epoxidation
Source: Sci Rep. 2018 Jan 26;8:1704. doi: 10.1038/s41598-018-19878-x (PMC5786050; doi:10.1038/s41598-018-19878-x)

Supporting Information

Chirality recognition of winding vine-shaped heterobiaryls with molecular asymmetry. Kinetic and dynamic kinetic resolution by Shi's asymmetric epoxidation

Kazuki Maruhashi, Yoichi Okayama, Ryo Inoue, Shiomi Ashida, Yuka Toyomori, Kentaro Okano, Atsunori Mori*

Department of Chemical Science and Engineering, Kobe University, 1-1 Rokkodai, Nada, 657-8501, Japan

Table of Contents

Experimental section ---s2
References ---s6
NMR spectra ---s7
HPLC profile ---s19

Experimental Section

General.

All the reactions were carried out under nitrogen atmosphere. ^{1}H NMR (300, 400 MHz) and ^{13}C NMR (100, 125 MHz) spectra were measured on JEOL ECZ400, Varian Gemini 300, or Bruker Avance 500 spectrometer. Unless noted, NMR spectra were measured at room temperature. The chemical shift was expressed in ppm with $CHCl_3$ (7.26 ppm for ^{1}H), $CDCl_3$ (77.0 ppm for ^{13}C) as internal standards. High resolution mass spectra (HRMS) were measured by JEOL JMS-T100LP AccuTOF LC-Plus (ESI) with a JEOL MS-5414DART attachment. For thin layer chromatography (TLC) analyses throughout this work, Merck precoated TLC plates (silica gel 60 F_{254}) were used. Purification by HPLC with preparative SEC column (JAI-GEL-2H) was performed by JAI LC-9201. HPLC by chiral column was performed with JASCO LC-2000 Plus using DAICEL Chiralpak IC or IF (0.46 mm id, 25 cm length) with the flow rate = 1.0 mL/min unless noted. Chemicals were purchased and used without further purification unless noted. Bisbenzoimidazole **1** was prepared according to the procedure described in our previous report.[1] Separation of racemic **1** by preparative HPLC with chiral column was carried out with DAICEL Chiralpak IF (20 mm id, 25 cm length). DFT calculation was carried out with Spartan ver 14 (Wavefunction Co. Ltd.). L-Menthone and D-Camphor were purchased and used without further purification. D-epoxone was purchased from Alfa-Aesar Co. Ltd. Other chiral ketones for Shi's asymmetric epoxidation were prepared according to the reported procedures.[2,3] Tetrabromobisimidazole (±)-**4a** was prepared by the procedure in our previous report.[4] Unsymmetrical heterbiaryls (±)-**4b**, (±)-**4c**, and (±)-**4e** were prepared by the procedures in our previous report.[5] Bithiophene (±)-**4d** was prepared by the procedure described in our previous report.[6] Racemic epoxides **3** and **5d** were prepared by epoxidation of (±)-**1** and (±)-**4d** with *m*-chloroperbenzoic acid or oxone/acetone in a manner described previously.[4]

Shi's asymmetric epoxidation of enantiopure bisbenzoimidazole (*S*a)-(+)-1: In a screw capped test tube were placed a buffer solution composed of 0.05 M $Na_2B_4O_7 \cdot 10H_2O$ in 4×10^{-4} M aqueous Na_2(EDTA) (0.7 mL), acetonitrile (1.5 mL), bisbenzoimidazole (S_a)-**1** (31.4 mg, 0.1 mmol), D-epoxone (0.06 mmol), and tetrabutylammonium hydrogen sulfate (1.5 mg, 0.004 mmol). The reaction mixture was warmed to 40 °C and a solution of Oxone (166 mg, 0.27 mmol) in aqueous Na_2(EDTA) (4 x 1.0 mL) and aqueous solution of K_2CO_3 (160 mg, 1.16 mmol in 1.0 mL of water) were added successively in three portions with each 1 h interval. The reaction mixture was stirred at 40 °C for an additional 24 h. The reaction mixture was diluted with water and extracted twice with ethyl acetate. The combined organic layer was washed with brine, dried over anhydrous sodium acetate, and concentrated under reduced pressure to leave a crude oil. The enantioselectivity and conversion of the reaction was estimated by HPLC analysis with a chiral column (DAICEL Chiralpak IF) using hexane/ethanol = 1:1 as an eluent to show 50% conversion to **3** whose enantiomeric ratio was revealed as >99:1 (t_R = 15.0 min).

Shi's asymmetric epoxidation of enantiopure bisbenzoimidazole (*R*a)-(−)-1: The reaction was carried out in a similar manner under similar conditions as described above to result in 3% conversion by HPLC analysis with chiral column (DAICEL Chiralpak IF) to confirm recovery of unreacted **(*R*a)-1** with e. r. of >99:1 (t_R = 8.8 min) using

hexane/ethanol = 1:1 as an eluent.

Kinetic resolution of racemic bisbenzoimidazole (±)-1 by Shi's asymmetric epoxidation: The reaction was carried out in a similar manner to that of **(*S*a)-(+)-1** to show HPLC profile of t_R = 6.4 min ((*S*a)-**1**), 8.6 min ((*R*a)-**1**), 11.5 min (**3**), and 13.9 min (**3**), respectively, with a chiral column (DAICEL Chiralpak IF) using hexane/ethanol = 1:1 as an eluent.

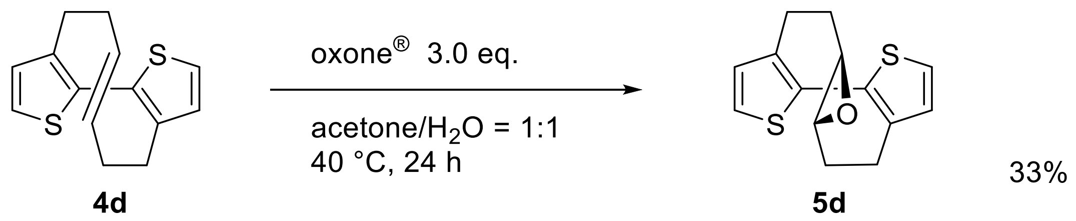

Epoxidation of racemic bithiophene (±)-**4d:** The reaction was carried out in a similar manner to the synthesis of **3** with **4a**(106 mg, 0.2 mmol) and oxone® (369 mg, 0.6 mmol) in acetone (0.8 mL)/water (0.8 mL) at 40 °C for 66 h to afford 46 mg of epoxide **5a** (33% yield). HPLC analysis with a chiral column (Daicel Chiralpak IF, flow rate = 1.0 mL/min) using hexane/ethanol=100:1 as an eluent indicated separation at the retention times of 11.0 and 12.0 min. ^{1}H NMR (300 MHz, $CDCl_3$) δ 0.88-1.02 (m, 2H), 2.00 (dt, *J* = 11.6, 2.2 Hz, 2H), 2.26-2.36 (m, 2H), 2.69-2.87 (m, 4H), 6.91 (d, *J* = 5.2 Hz, 2H), 7.31 (d, *J* = 5.2 Hz, 2H) ; ^{13}C NMR (125 MHz, $CDCl_3$) δ 24.16, 33.94, 57.94, 126.04, 129.33, 129.63, 142.35; IR (ATR) 2963, 2923, 2856, 1469, 1444, 1413, 1236, 1215, 1093, 1042, 1023, 984, 906, 863, 854, 822, 797, 782, 732, 691, 677, 660 cm-1; HRMS (DART-ESI+) Calcd. for $C_{14}H_{14}S_2O[M+H]^+$: 263.05643; found: m/z 263.05724.

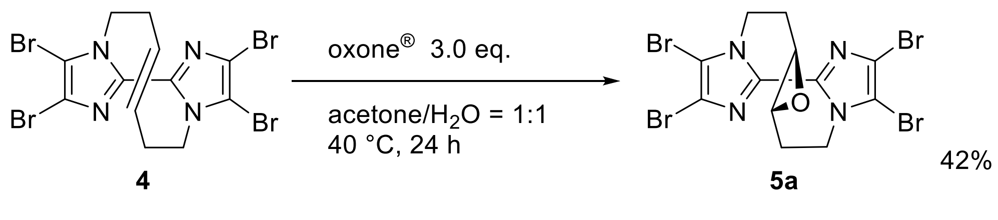

Epoxidation of racemic tetrabromobisimidazole (±)-4a: The reaction was carried out in a similar manner to the synthesis of **3** with **4a**(106 mg, 0.2 mmol) and oxone® (369 mg, 0.6 mmol) in acetone (0.8 mL)/water (0.8 mL) at 40 °C for 66 h to afford 46 mg of epoxide **5a** (42% yield), which was purified by column chromatography on silica gel using hexane/methyl acetate = 5:1-3:1 as an eluent. HPLC analysis with chiral column (DAICEL Chiralpak IF) using hexane/ethanol = 1:1 as an eluent indicated separation at the retention times of 8.8 and 14.7 min, respectively. ^{1}H NMR (300 MHz) δ 1.13 (dddd, *J* = 13.7, 14.0, 10.1, 3.7 Hz, 1.0H), 1.79 (dd, *J* = 9.6, 2.7 Hz, 1.0H), 2.36 (dd, 13.7, 1.8 Hz, 1.0H), 4.18 (dt, 15.1, 3.4 Hz, 1.0H), 4.73 (ddd, *J* = 15.2, 13.6, 1.8 Hz, 1.0H); ^{13}C NMR (125 MHz) δ 32.8, 43.2, 53.7, 106.2, 117.3, 137.8; IR (ATR): 2968, 2924, 2853, 1745, 1481, 1456, 1439, 1416, 1394, 1363, 1337, 1311, 1229, 1121, 992, 957, 755, 736 cm^{-1}; HRMS (DART+) Calcd. for $C_{12}H_{11}{}^{79}Br_4N_4O$ $[M + H]^+$: 542.76664, found: *m/Z* 542.76780.

Epoxidation of racemic heterobiaryl composed of benzoimidazole and bisimidazole (±)-4b:

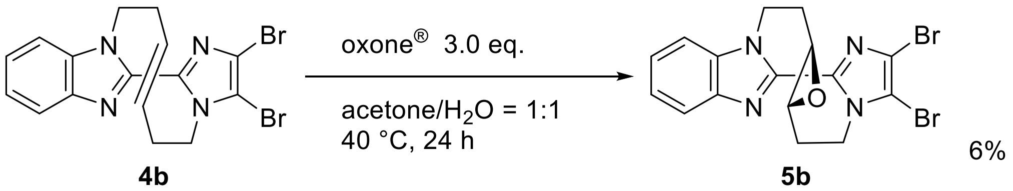

Epoxidation of racemic (±)-**4b** was carried out in a similar manner as described above. (6% yield). HPLC analysis with a chiral column (DAICEL Chiralpak IF, flow rate = 1.0 mL/min) using hexane/ethanol = 1:1 as an eluent of epoxide indicated separation at the retention times of 11.4 and 14.5 min. ^{1}H NMR (300 MHz) δ 1.07-1.24 (m, 2.3H), 1.49 (dt, *J* = 9.6, 2.7 Hz, 1.1H), 1.88 (dt, *J* = 9.2, 2.5 Hz, 1.0H), 2.28 (d, *J* = 12.8 Hz, 0.9H), 2.39 (d, *J* = 12.8 Hz, 1.0H), 4.24 (dt, *J* = 14.6, 3.2 Hz, 1.0H), 4.43 (dt, *J* = 15.6, 3.4 Hz, 1.1H), 4.84-5.01 (m, 1.8H), 7.33-7.43 (m, 3.1H), 7.85 (d, *J* = 6.4 Hz, 1.1H); ^{13}C NMR (125 Hz) δ 32.5, 33.0, 40.3, 43.4, 53.9, 54.7, 106.5, 110.0, 117.5, 120.7, 123.5, 124.5, 133.8, 139.0, 142.3, 142.5; IR (ATR): 2964, 2926, 1745, 1728, 1481, 1455, 1424, 1406, 1229, 1098, 1024, 798, 750 cm^{-1}; HRMS (DART+) Calcd. for $C_{16}H_{15}{}^{79}Br_2N_4O$ [M + H]$^+$: 436.96126, found: *m/z* 436.96110.

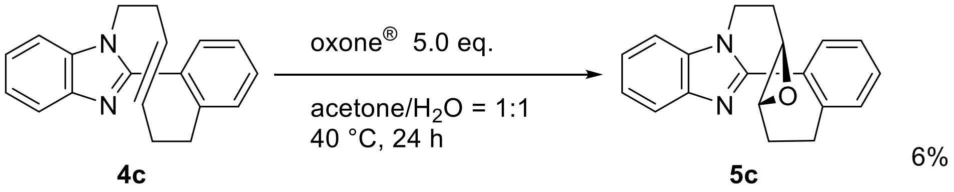

Epoxidation of racemic heterobiaryl (±)-4c: Epoxidation of racemic (±)-**4c** was carried out in a similar manner to that of (±)-**4d** to afford racemic **5d** in 6% yield. HPLC analysis with a chiral column (DAICEL Chiralpak IF, flow rate = 1.0 mL/min) using hexane/ethanol = 1:1 as an eluent of epoxide indicated separation at the retention times of 6.1 and 6.8 min. ^{1}H NMR (400 MHz) δ 0.89-1.00 (m, 1.2H), 1.28-1.40 (m, 1.2H), 1.53 (dt, *J* = 10.1, 2.7 Hz, 0.9H), 1.97 (dt, *J* = 10.1, 2.9 Hz, 1.0H), 2.30 (d, *J* = 12.9 Hz, 1.0H), 2.34-2.41 (m, 1.1H), 2.77 (ddd, *J* = 13.7, 5.5, 2.3 Hz, 0.9H), 3.50 (td, *J* = 14.0, 2.3 Hz, 0.9H), 4.28-4.44 (m, 2.0H), 7.28-7.41 (m, 5.8H), 7.46 (td, *J* = 7.32, 1.4 Hz, 1.0H), 7.85 (t, *J* = 4.6 Hz, 1.0H); ^{13}C NMR (100 Hz) δ 28.2, 32.4, 34.6, 40.2, 55.7, 56.0, 110.0, 120.0, 122.7, 123.0, 126.2, 129.5, 129.8, 130.4, 131.0, 133.7, 142.4, 142.8, 153.1; IR (ATR): 2961, 2924, 2854, 1745, 1727, 1455, 1386, 1361, 1260, 1088, 1028, 797, 748 cm^{-1}; HRMS (DART+) Calcd. for $C_{19}H_{19}N_2O_1$ [M + H]$^+$: 291.14974, found: *m/z* 291.15056.

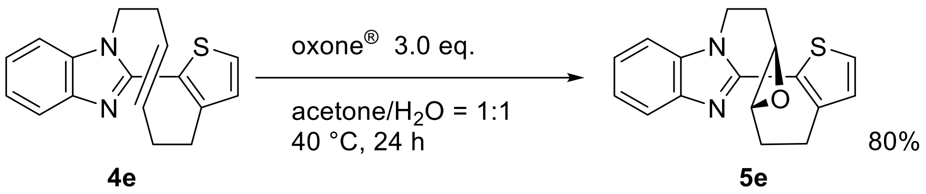

Epoxidation of racemic (±)-4e: The reaction was carried out in a similar manner to the synthesis of **5b** with (±)-**4c** (100 mg, 0.36 mmol), oxone® (1.1 g, 1.8 mmol) in acetone (1.5 mL) and water (1.5 mL) at 40 °C for 72 h to afford 5.8 mg of corresponding epoxide **5c** (6% yield), which was purified by column chromatography on silica gel using hexane/methyl acetate = 1:1 as an eluent. HPLC analysis with a chiral column (DAICEL Chiralpak IF, flow rate = 0.5 mL/min) using hexane/ethanol = 10:1 as an eluent of epoxide indicated separation at the retention times of 68.3 and 74.6 min. ^{1}H NMR (400 MHz) δ 0.89-1.01 (m, 1.0H) 1.24-1.38 (m, 1.4H), 1.68 (d, J = 9.6 Hz, 1.1H), 2.04 (d, J = 10.1 Hz, 0.9H), 2.31 (d, J = 11.9 Hz, 0.9H), 2.38 (d, J = 13.7 Hz, 1.0H), 2.86 (d, J = 12.8 Hz, 1.1H), 3.14 (t, J = 13.3 Hz, 1.0H), 4.42 (d, J = 15.1 Hz, 0.9H), 4.56 (t, J = 14.0 Hz, 0.9H), 7.03 (d, J = 5.0 Hz, 1.0H), 7.34 (s, 2.8H), 7.48 (d, J = 5.0 Hz, 1.0H), 7.87 (s, 0.9 H); ^{13}C NMR (100 Hz) δ 23.9, 32.5, 33.2, 40.4, 56.3, 56.5, 109.4, 120.3, 122.7, 123.3, 125.2, 127.3, 129.5, 134.2, 142.9, 145.0, 147.43; IR (ATR): 3062, 2934, 2855, 1739, 1564, 1499, 1455, 1377, 1161, 1023, 986, 929, 871, 805, 746 cm^{-1}; HRMS (DART+) Calcd. for $C_{17}H_{16}N_2O_1S_1$ [M + H]$^+$: 297.10616, found: *m/z* 297.10578.

Kinetic resolution of racemic (±)-4d by Shi's epoxidation: The reaction was carried out in a similar manner for the resolution of (±)-**1**. When the reaction was carried out with chiral ketone **2e** to undergo the reaction at 39% conversion. Epoxide **5d** was obtained with er of 96:4, which was confirmed by HPLC analysis with chiral column. (DAICEL Chiralpak IF, eluent: hexane/ethanol = 100:1, t_{R} = 10.9 and 11.9 min, flow rate = 1.0 mL/min) with recovery of **4d** as a mostly racemic mixture (ca. 50:50, t_R = 12.0 min and 13.4 min, eluent: hexane, flow rate = 0.5 mL/min).

Kinetic resolution of (±)-**4a**, (±)-**4b**, and (±)-**4c** was carried out in the above manner. (±)-**4a**: t_R = 6.3 min, t_R = 10.4 min; **5a**: t_R = 8.6 min, t_R = 12.8 min, respectively, with chiral column. (DAICEL Chiralpak IF, eluent: hexane/ethanol = 1:1, flow rate = 1.0 mL/min. (±)-**4b**: t_R = 6.8 min, t_R = 8.8 min; **5a**: t_R = 11.6 min, t_R = 13.0 min, respectively, with chiral column. (DAICEL Chiralpak IF, eluent: hexane/ethanol = 1:1, flow rate = 1.0 mL/min. (±)-**4c**: t_R = 4.5 min; **5c**: t_R = 6.0 min, t_R = 6.6 min, respectively, with chiral column. (DAICEL Chiralpak IF, eluent: hexane/ethanol = 1:1, flow rate = 1.0 mL/min and t_R = 90 min and 93 min, respectively, with DAICEL Chiralpak IF, eluent: hexane/ethanol = 50:1, flow rate = 0.5 mL/min to show the ratio of ca. 96:4 (by curve fitting).

Dynamic Kinetic resolution of racemic bithiophene (±)-**4e by Shi's epoxidation:** The reaction was carried out in a similar manner as described above. When chiral ketone **2e** was employed for the reaction, HPLC analysis with chiral column (DAICEL Chiralpak IF, flow rate = 0.5 mL/min) using hexane/ethanol = 10:1 as an eluent for unreacted **4e** revealed to exhibit 4% recovery (t_R = 24.0 min, 29.3 min: 50:50 er) and epoxide **5e** with the enantiomeric ratio of 83:17 (t_R = 65.2 min, 72.1 min: 83:17 er).

Table of results on racemization barrier by experimental and calculated values

compound	$\Delta G^{\ddagger}$ (exp.) $kJmol^{-1}$	temp, K (solvent)[a]	$\Delta G^{\ddagger}$ (calcd.) $kJmol^{-1}$ [b]
**1**	130.15	373.15 (C_6H_5Cl)	138.58
**4a**	140.14	413.15 (C_6H_5Cl)	139.41
**4b**			138.29
**4c**			145.93
**4d**	101.69	313.15 (hexane)	106.15
**4e**			86.43

[a] Racemization barrier based on experiment with 1 mg/mL solution. See ref 4. [b] DFT calculations in a B3LYP/6-31G level.

References

(1) Nishio, S. *et al.* Axially Chiral Macrocyclic E -Alkene Bearing Bisazole Component Formed by Sequential C–H Homocoupling and Ring-Closing Metathesis. *Org. Lett.* **14,** 2476–2479 (2012).

(2) Goeddel, D. *et al.* Effective Asymmetric Epoxidation of Styrenes by Chiral Dioxirane. *J. Org. Chem.* **71,** 1715–1717 (2006).

(3) Tu, Y.; Wang, Z. X.; Frohn, M.; He, M.; Yu, H.; Tang, Y.; Shi, Y. *J. Org. Chem.* **1998**, *63*, 8475.

(4) Okayama, Y., Maruhashi, K., Tsuji, S. & Mori, A. Studies on Diastereoselective Functionalization, Optical Resolution, and Racemization Behaviors of Macrocyclic Bisimidazole of Winding-Vine-Shaped Molecular Asymmetry. *Bull. Chem. Soc. Jpn.* **88,** 1331–1337 (2015).

(5) Mori, A. *et al.* Synthesis of Unsymmetrical Heterobiaryls with Winding Vine-Shaped Molecular Asymmetry through a Condensation Pathway. *Heterocycles* **95,** 268 (2017).

(6) Toyomori, Y. *et al.* Bithiophene with Winding Vine-shaped Molecular Asymmetry. Preparation, Structural Characterization, and Enantioselective Synthesis. *Bull. Chem. Soc. Jpn.* **89,** 1480–1486 (2016).

^{1}H NMR of epoxide 3 derived from heterobiaryl 1

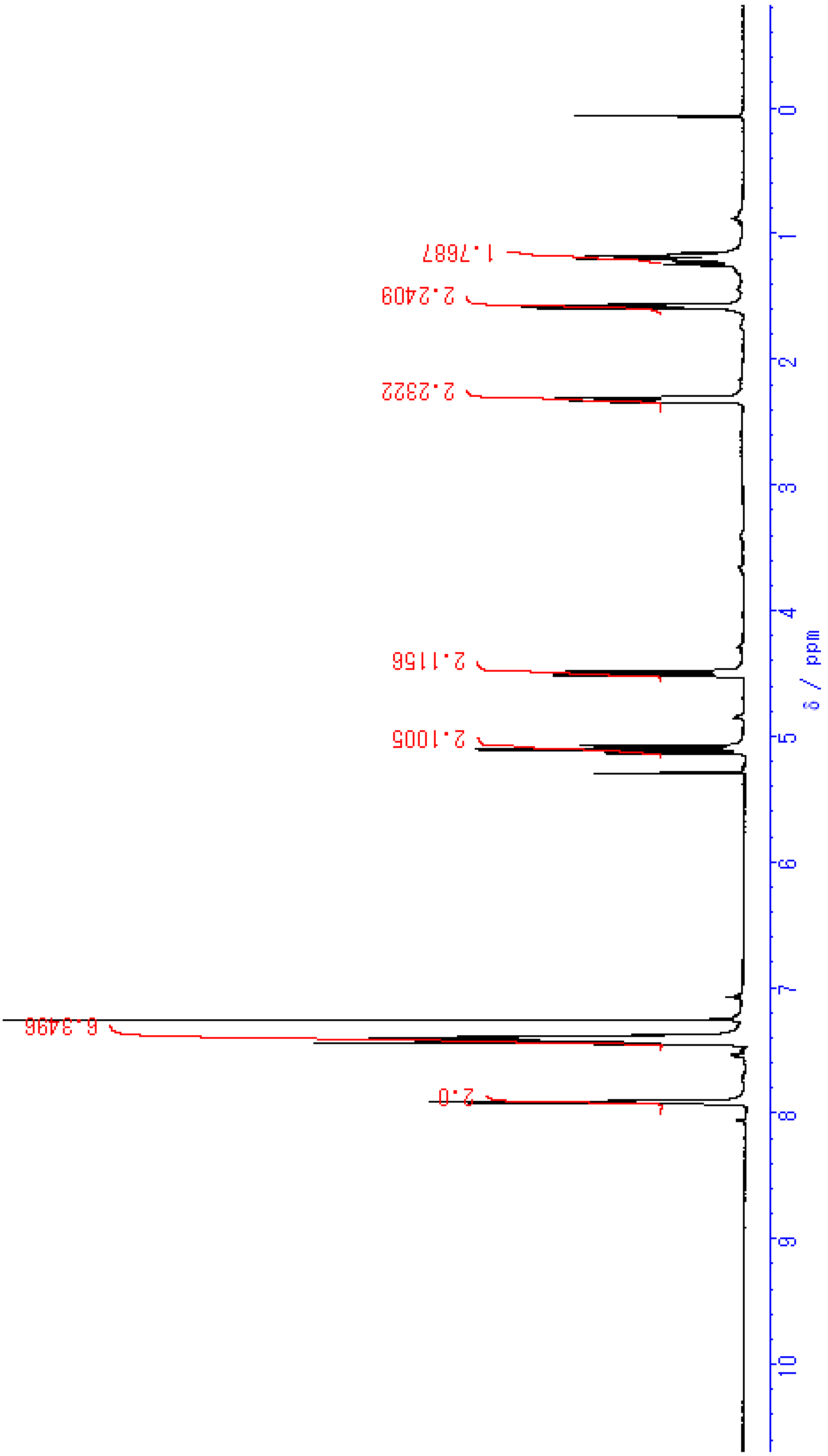

^{13}C NMR of epoxide 3 derived from heterobiaryl 1

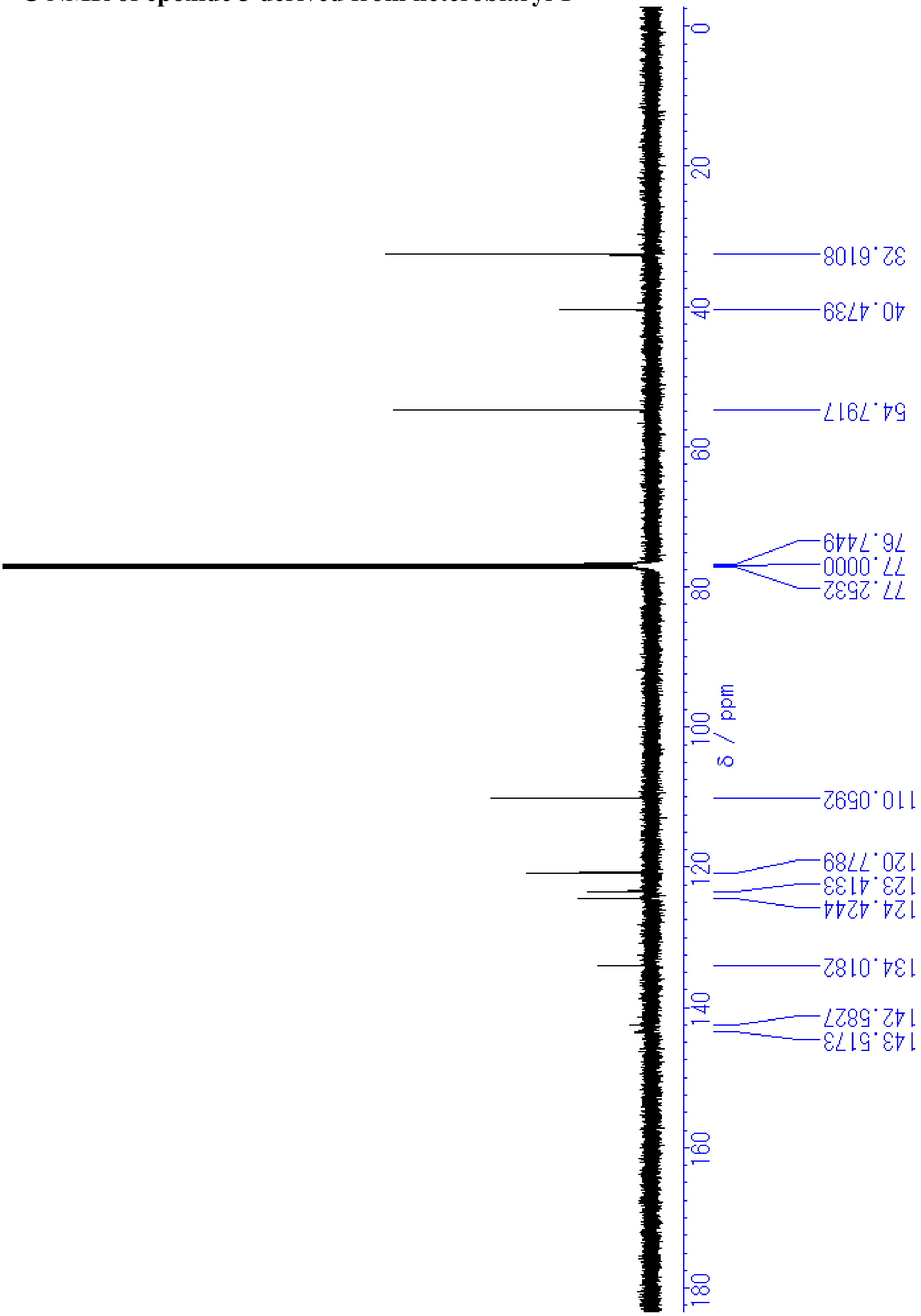

^{1}H NMR of epoxide 5a derived from tetrabromobisimidazole 4a

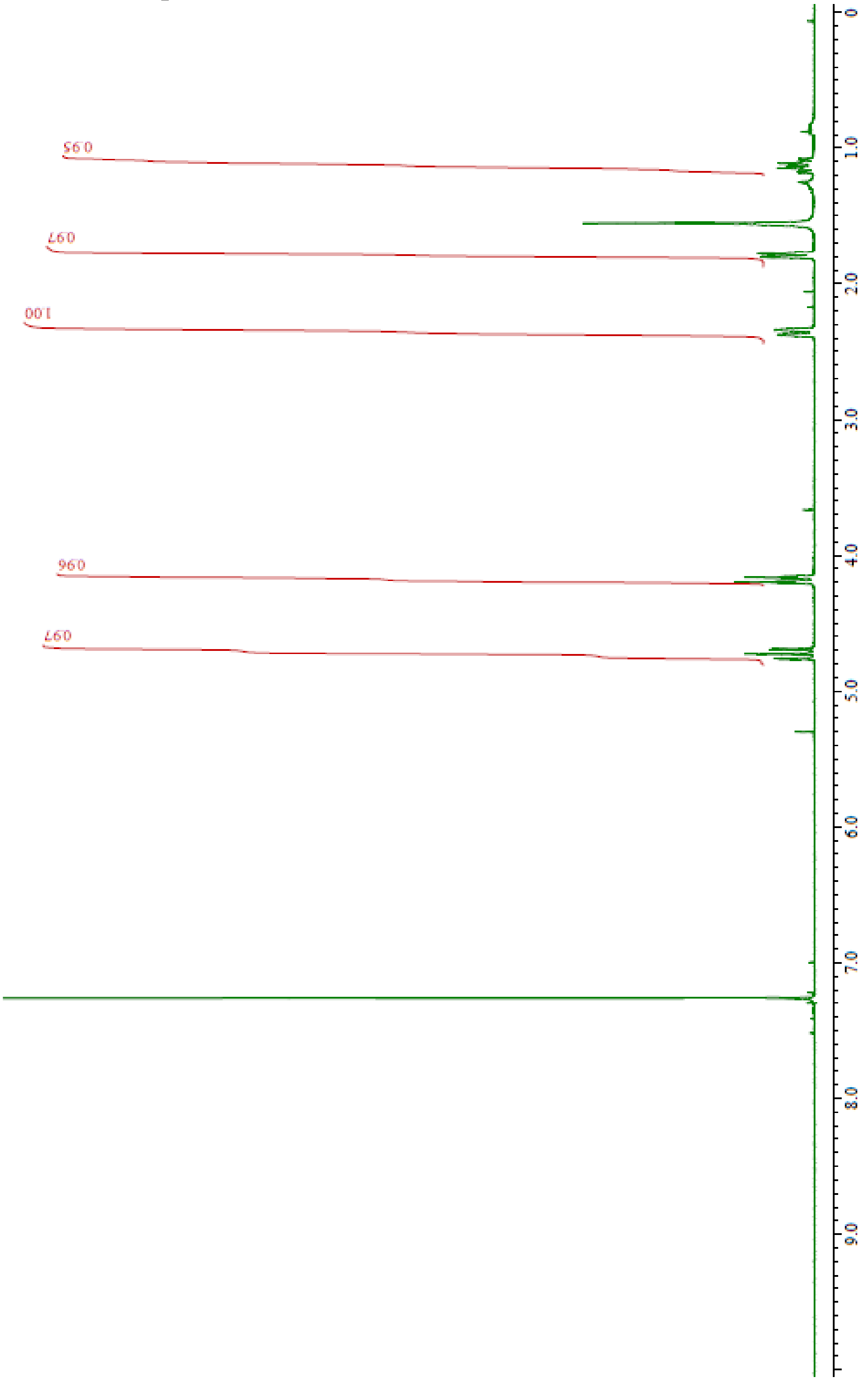

^{13}C NMR of epoxide 5a derived from tetrabromobisimidazole 4a

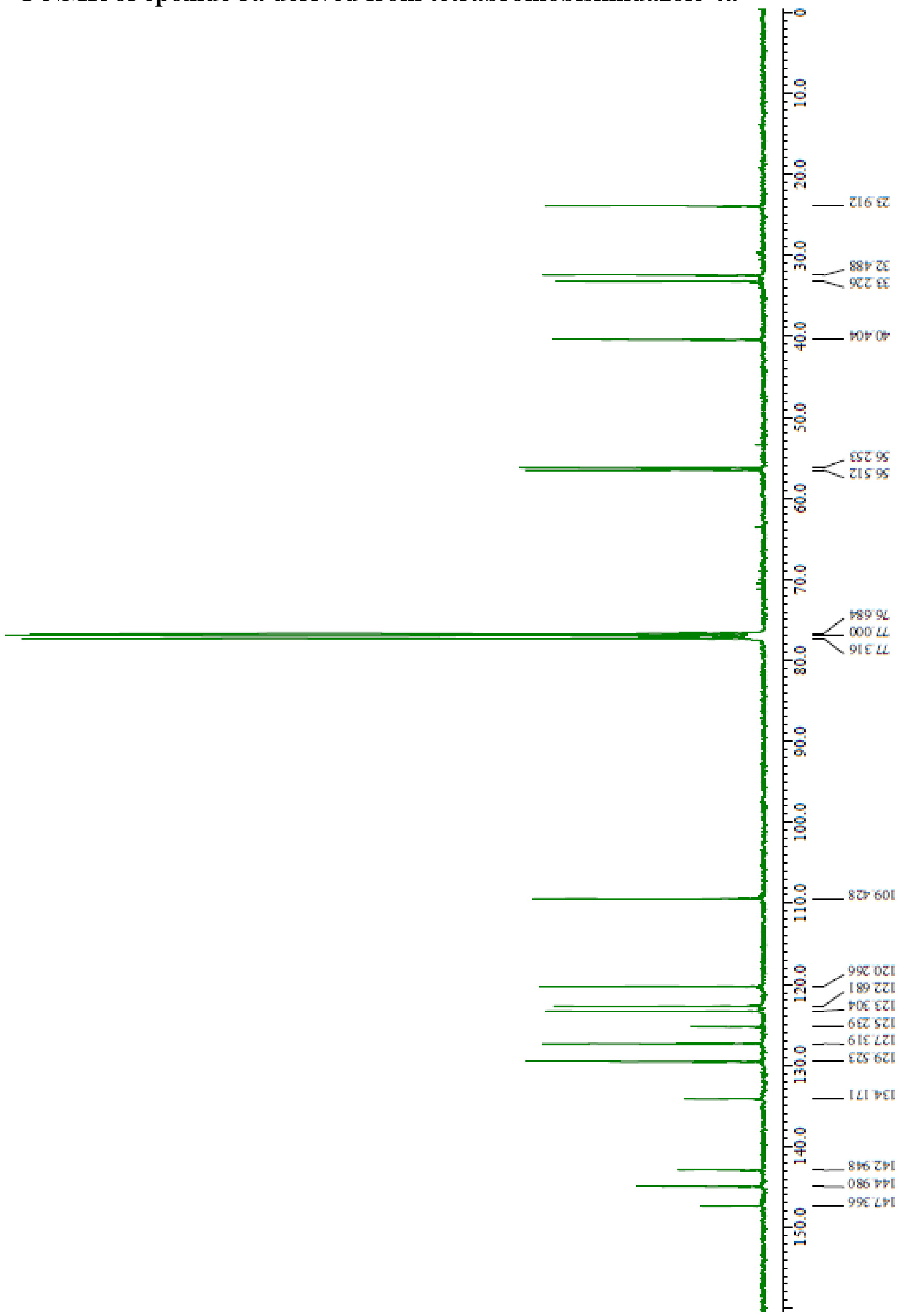

^{1}H NMR of epoxide 5b derived from heterobiaryl 4b (benzoimidazole & dibromoimidazole)

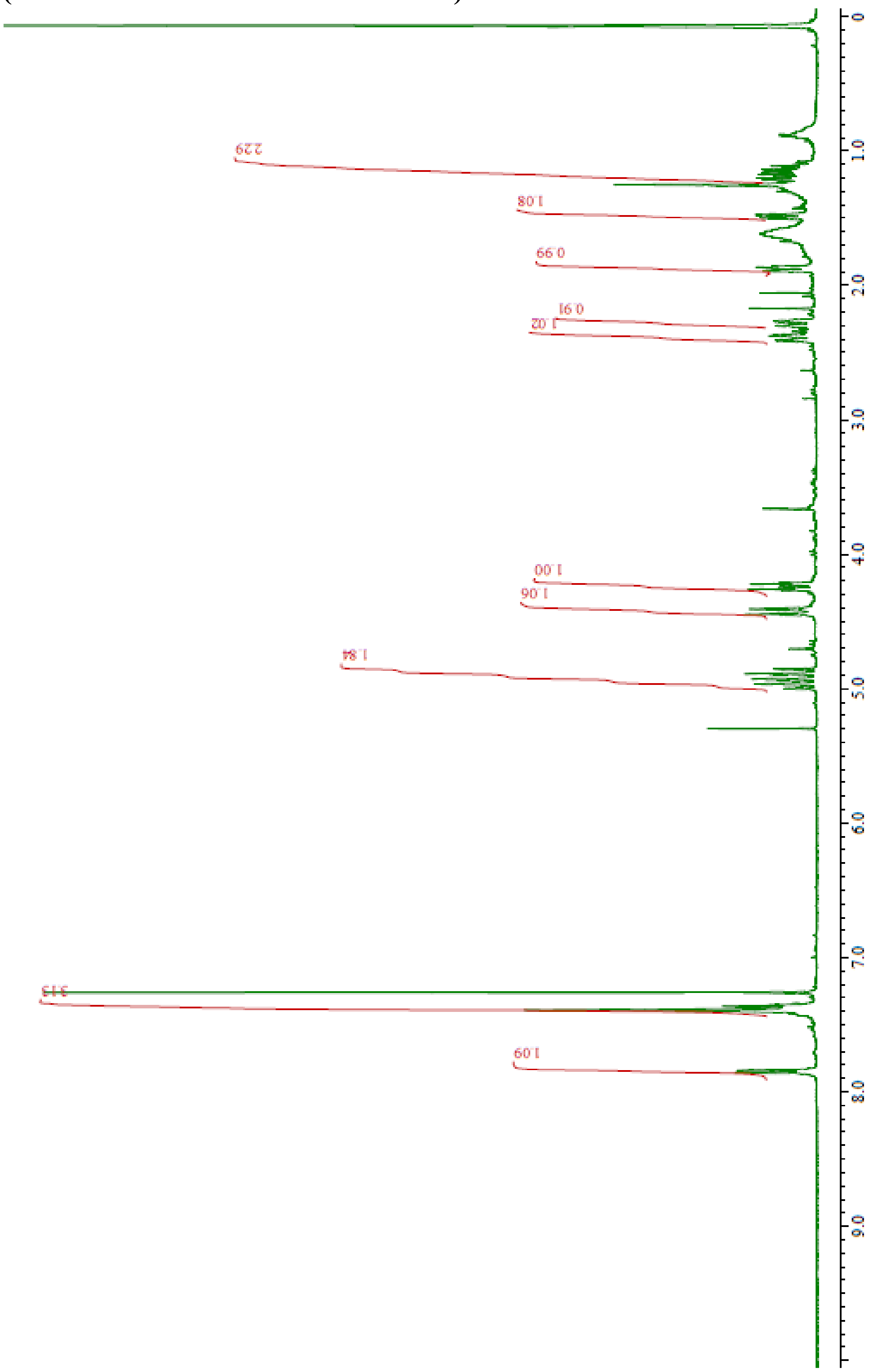

^{13}C NMR of epoxide 5b derived from heterobiaryl 4b (benzoimidazole & dibromoimidazole)

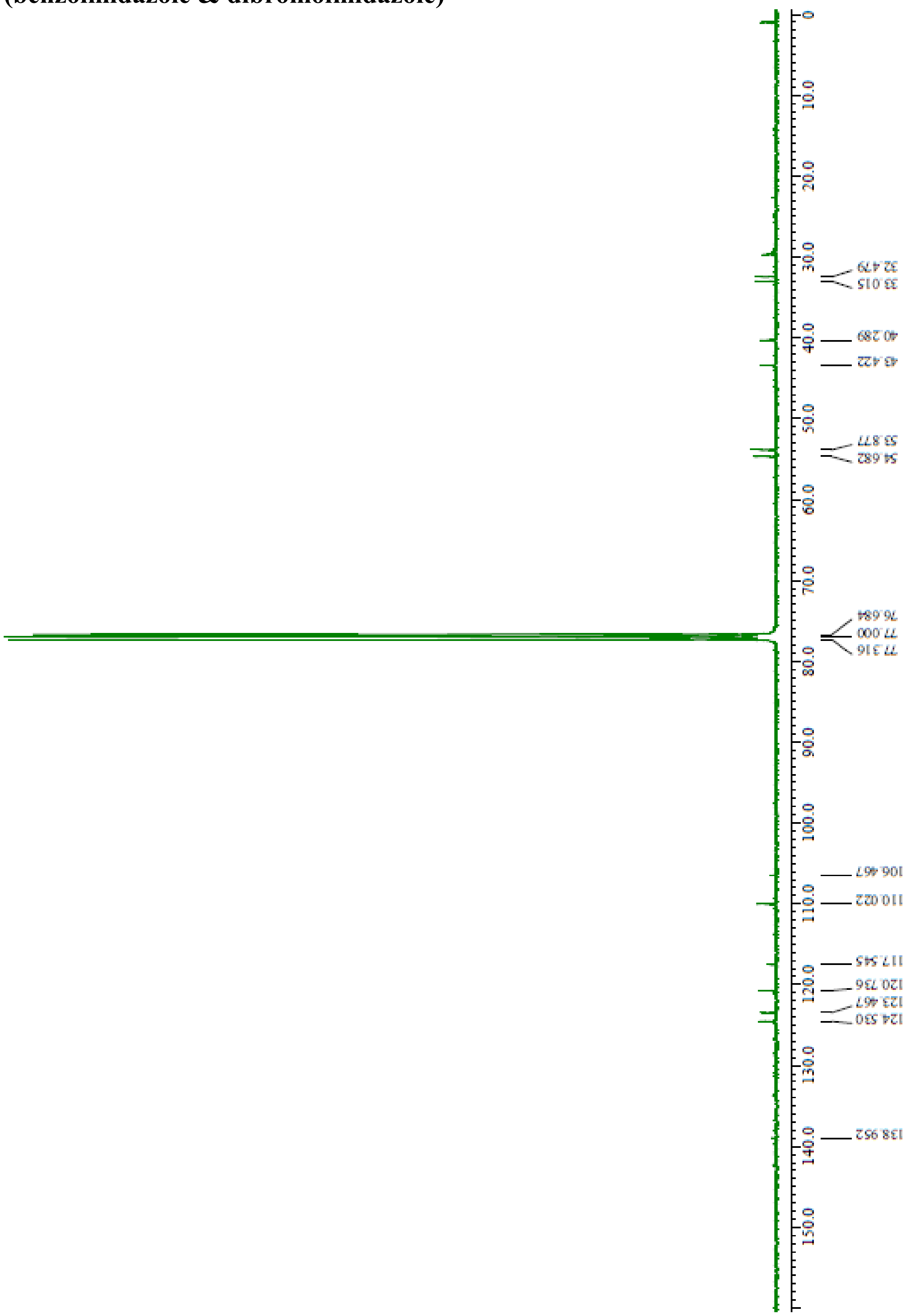

^{1}H NMR of epoxide 5c derived from heterobiaryl 4c (benzoimidazole & benzene)

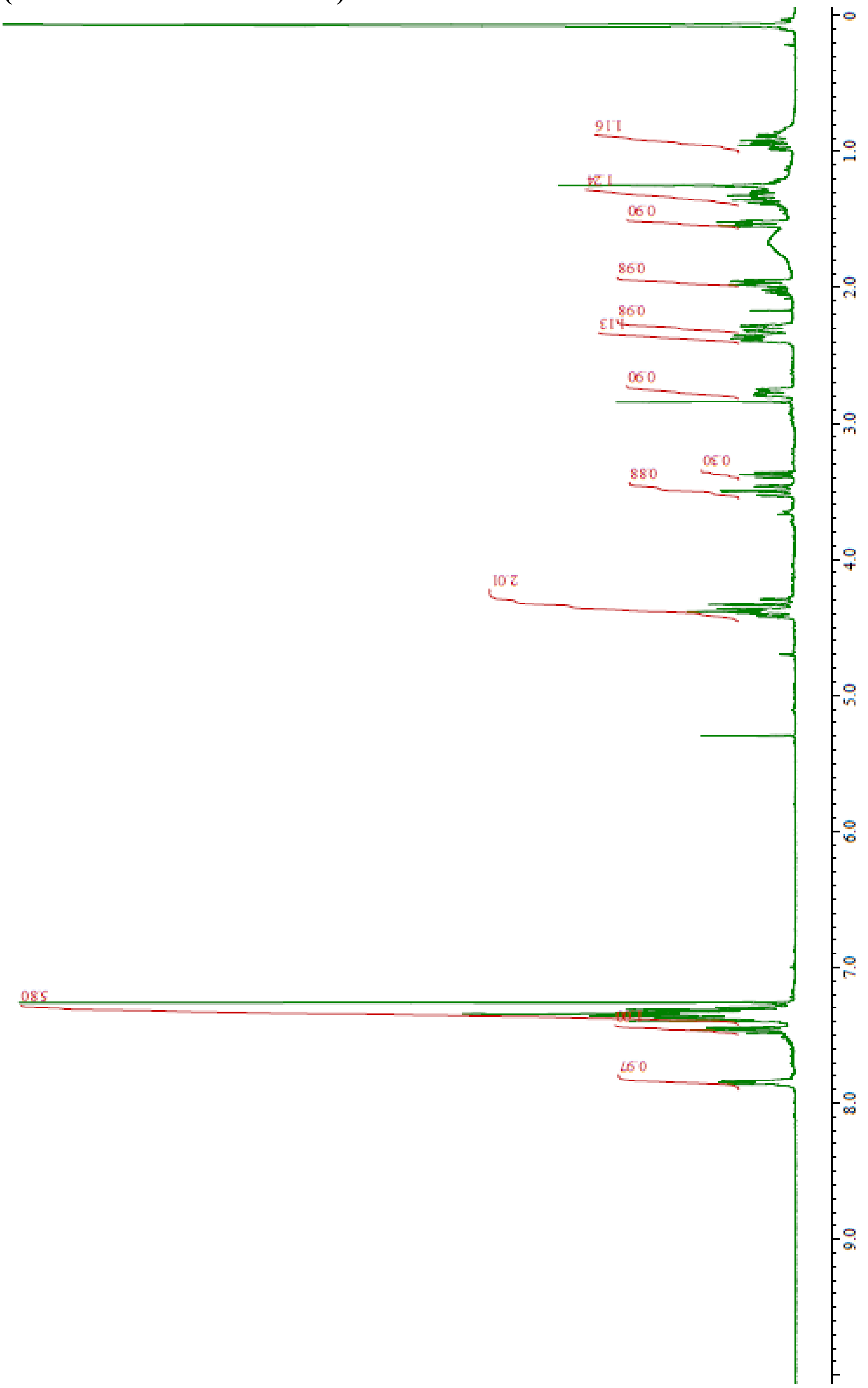

^{13}C NMR of epoxide 5c derived from heterobiaryl 4c (benzoimidazole & benzene)

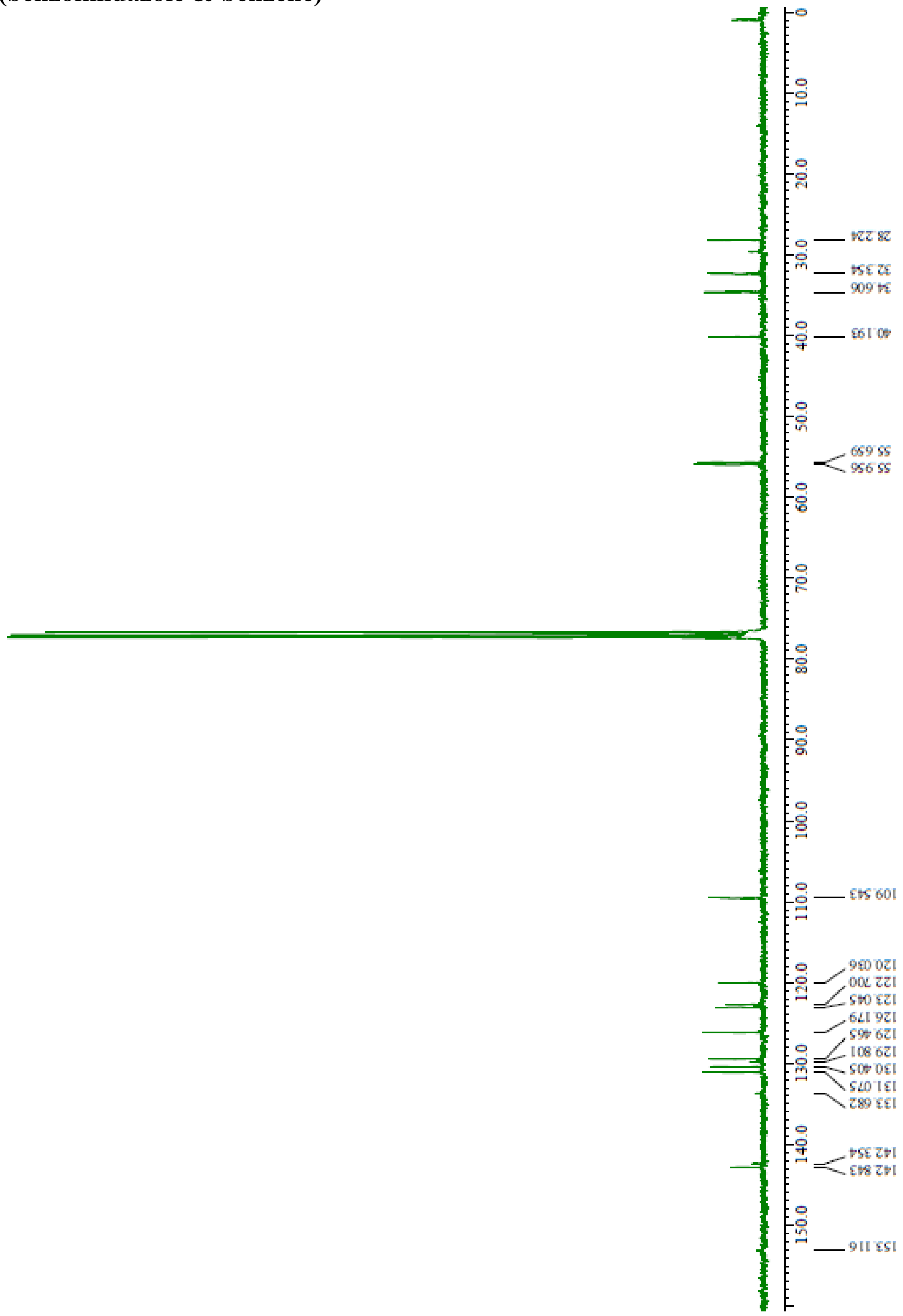

^{1}H NMR of epoxide 5d derived from bithiophene 4d

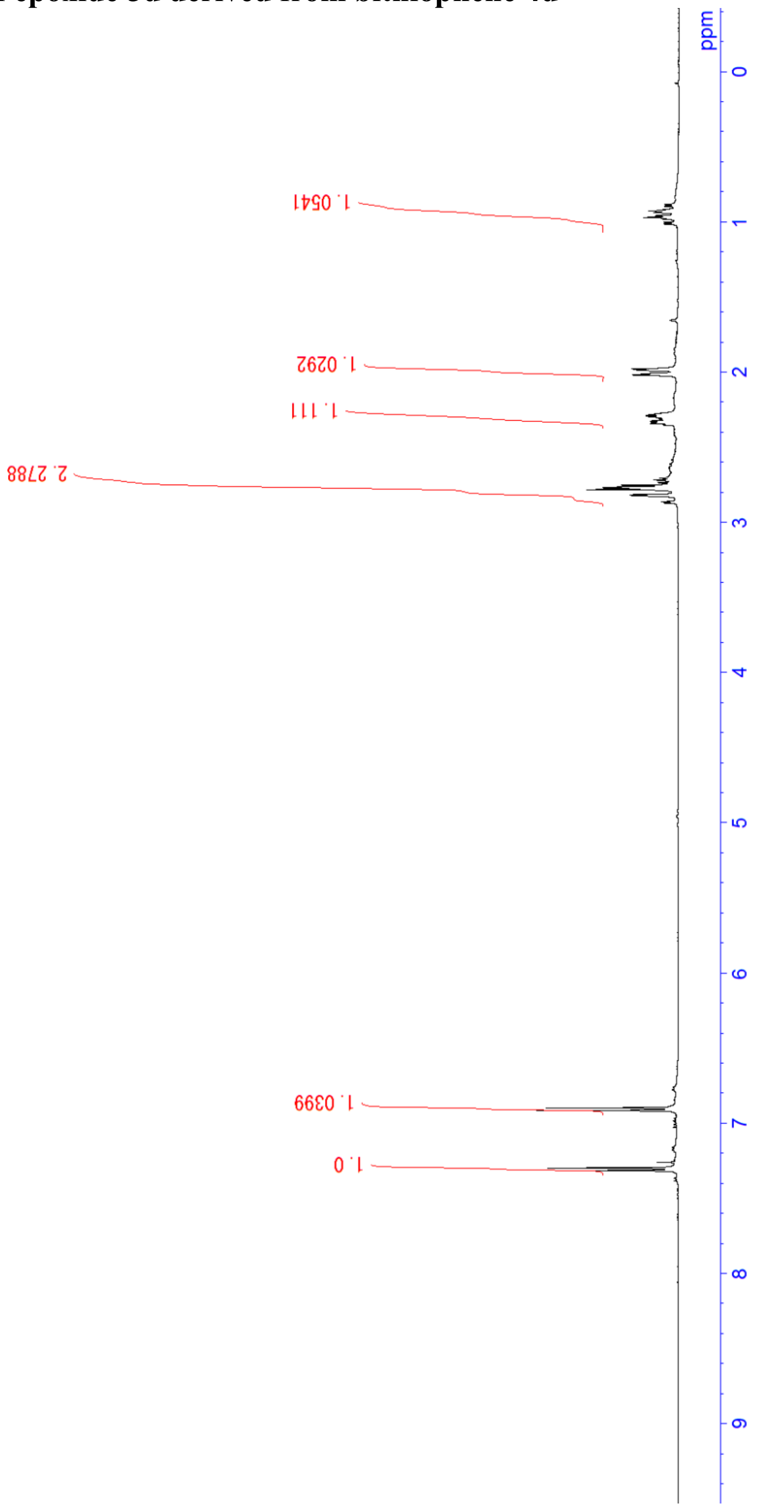

^{13}C NMR of epoxide 5d derived from bithiophene 4d

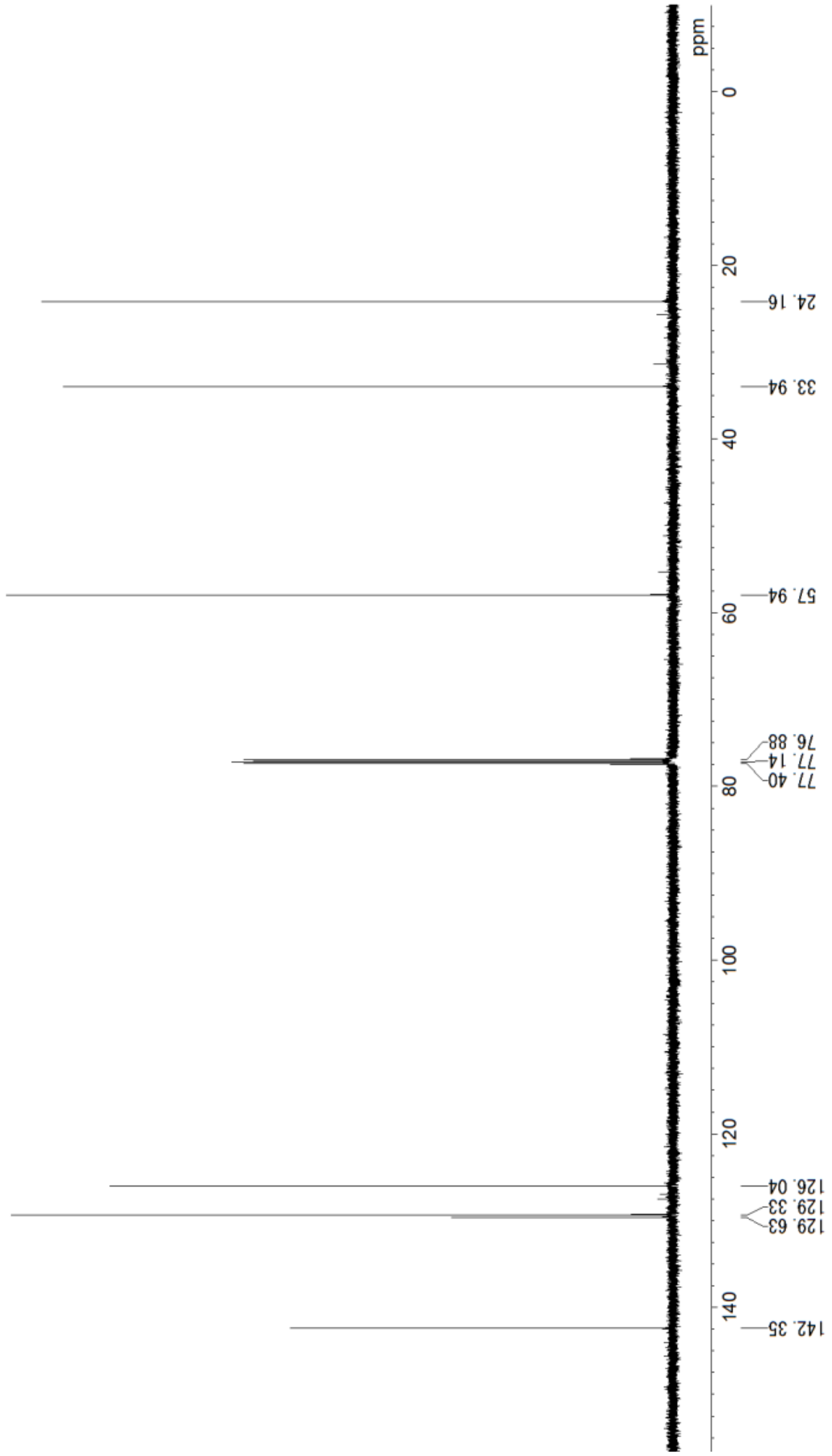

^{1}H NMR of epoxide 5e derived from heterobiaryl 4e (Benzoimdazole & thiophene)

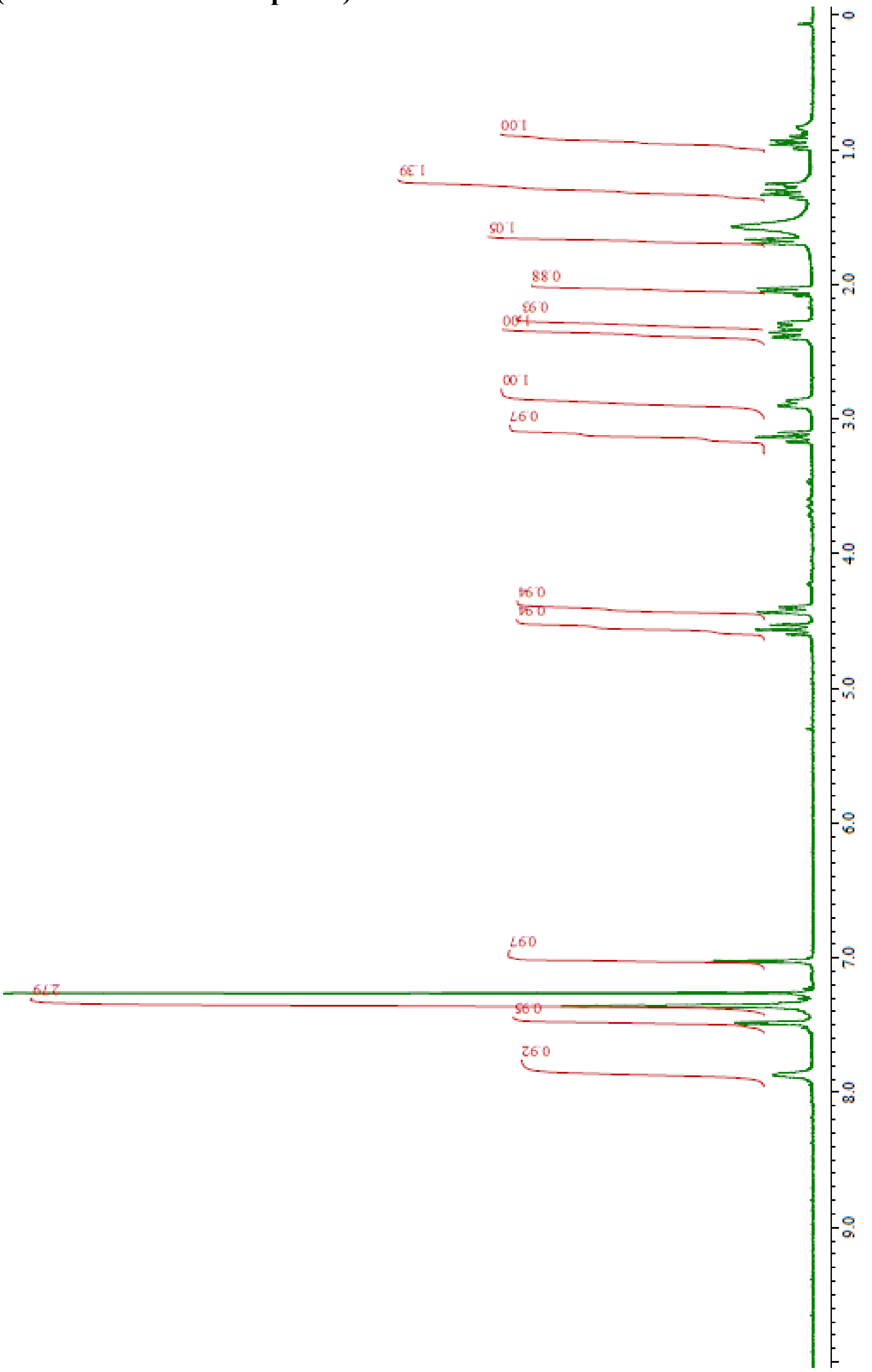

^{13}C NMR of epoxide 5e derived from heterobiaryl 4e (Benzoimdazole & thiophene)

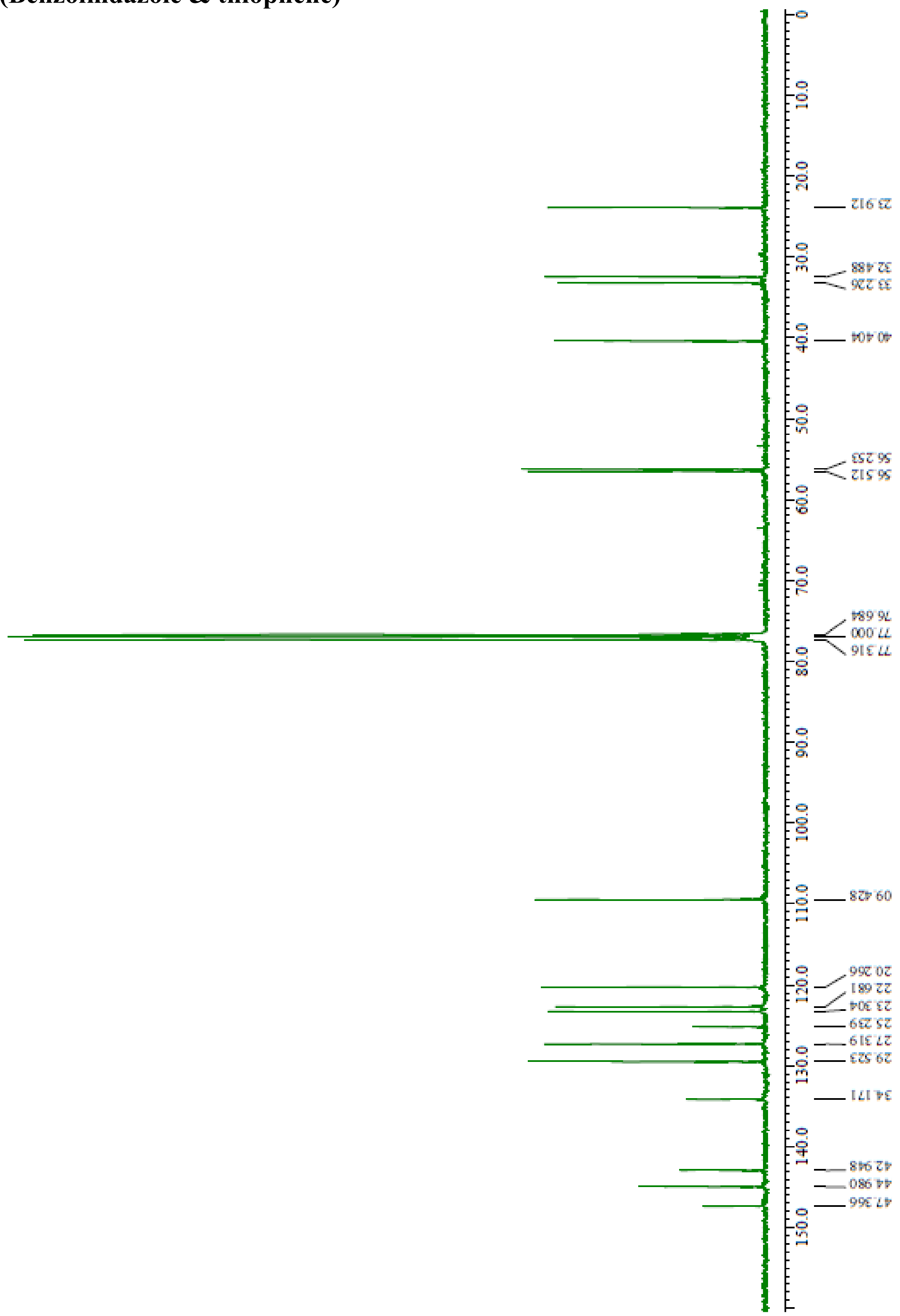

HPLC profile of (S_a)-1 (Chiralpak IF, eluent: hexane:ethanol = 1:1)
Flow rate = 1.0 mL/min

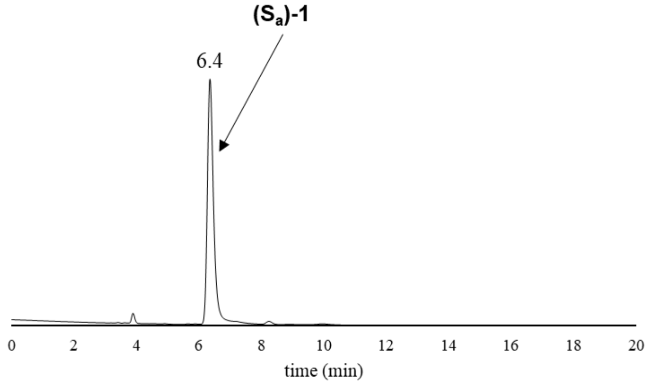

HPLC profile of (R_a)-1 (Chiralpak IF, eluent: hexane:ethanol = 1:1)
Flow rate = 1.0 mL/min

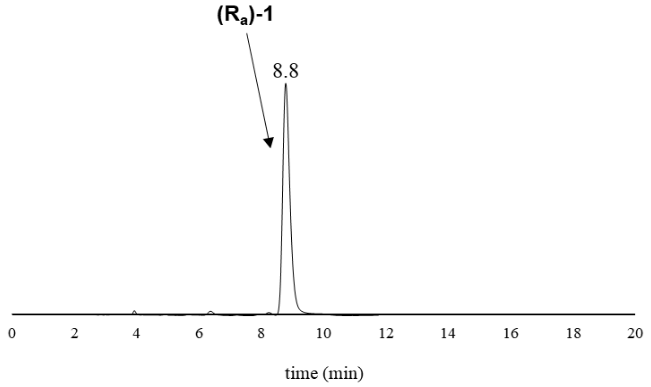

HPLC profile of racemic 1 (Chiralpak IF, eluent: hexane:ethanol = 1:1)
Flow rate = 1.0 mL/min

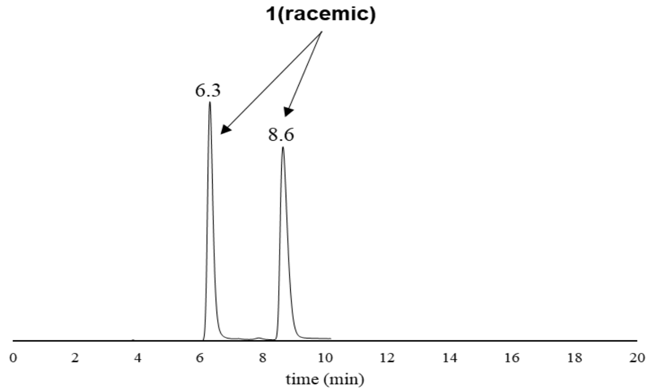

HPLC profile of racemic 3 (Chiralpak IF, eluent: hexane:ethanol = 1:1)
Flow rate = 1.0 mL/min

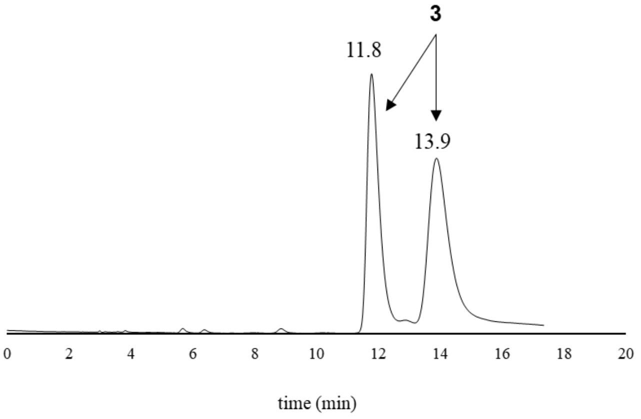

HPLC profile for the epoxidation product 3 from (S_a)-1 with D-epoxone (2a)
(Chiralpak IF, eluent: hexane:ethanol =1:1)
Flow rate = 1.0 mL/min

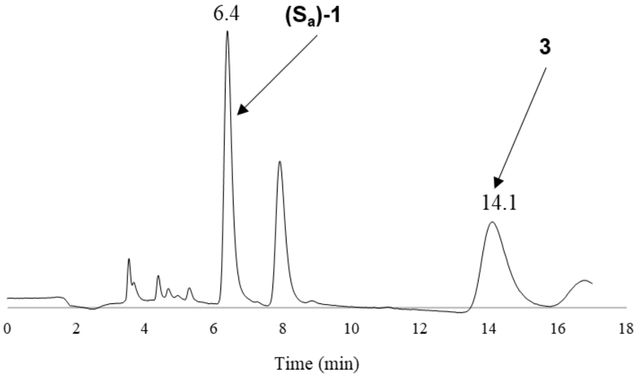

HPLC profile for the unreacted (R_a)-1 in the epoxidation with D-epoxone (2a)
(Chiralpak IF, eluent: hexane:ethanol =1:1)
Flow rate = 1.0 mL/min

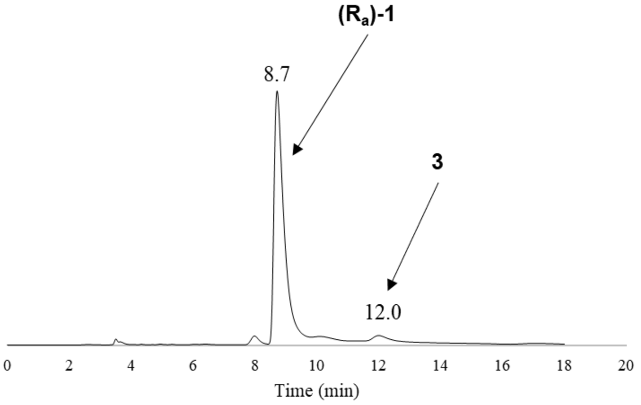

HPLC profile of the kinetic resolution of racemic 1 for the reaction shown in Table 1, entry 7 (Chiralpak IF, eluent: hexane:ethanol =1:1)
Flow rate = 1.0 mL/min

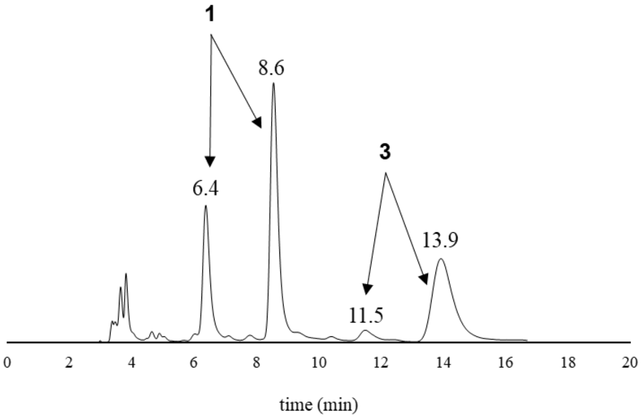

HPLC profile of racemic 4a (Chiralpak IF, eluent: hexane:ethanol = 1:1)
Flow rate = 1.0 mL/min

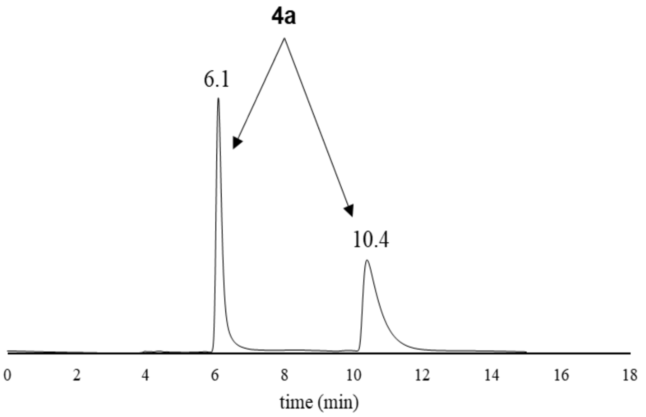

HPLC profile of racemic 5a (Chiralpak IF, eluent: hexane:ethanol = 1:1)
Flow rate = 1.0 mL/min

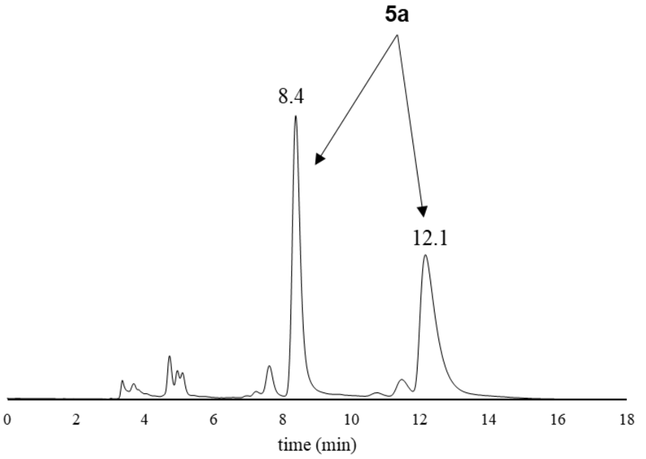

HPLC profile of the kinetic resolution of racemic 4a to give epoxide 5a (Chiralpak IF, eluent: hexane:ethanol =1:1)
Flow rate = 1.0 mL/min

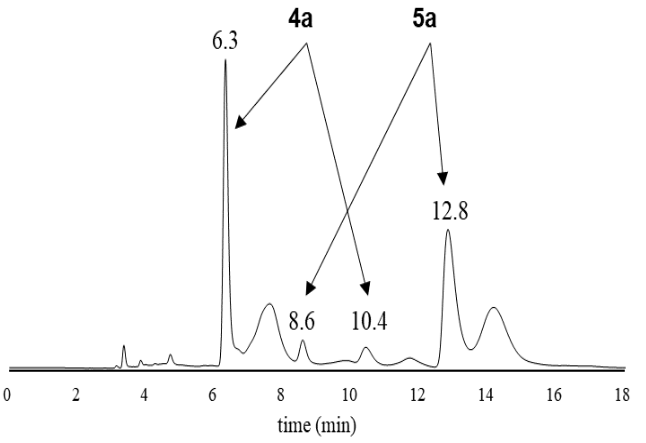

HPLC profile of racemic 4b (Chiralpak IF, eluent: hexane:ethanol = 1:1)
Flow rate = 1.0 mL/min

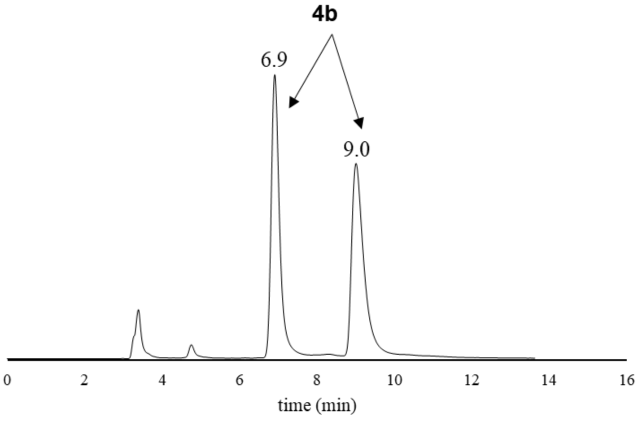

HPLC profile of racemic 5b (Chiralpak IF, eluent: hexane:ethanol = 1:1)
Flow rate = 1.0 mL/min

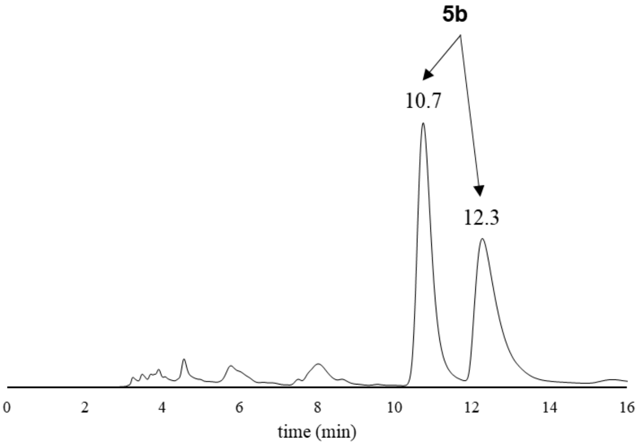

HPLC profile of the kinetic resolution of racemic 4b to give epoxide 5b (Chiralpak IF, eluent: hexane:ethanol =1:1)
Flow rate = 1.0 mL/min

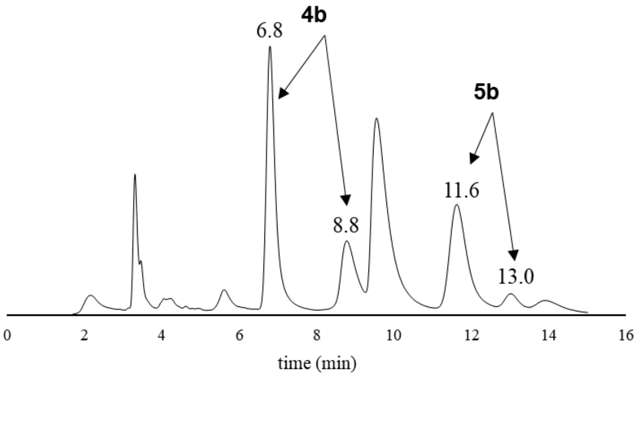

HPLC profile of racemic 4c (Chiralpak IF, eluent: hexane:ethanol = 50:1)
Flow rate = 0.5 mL/min

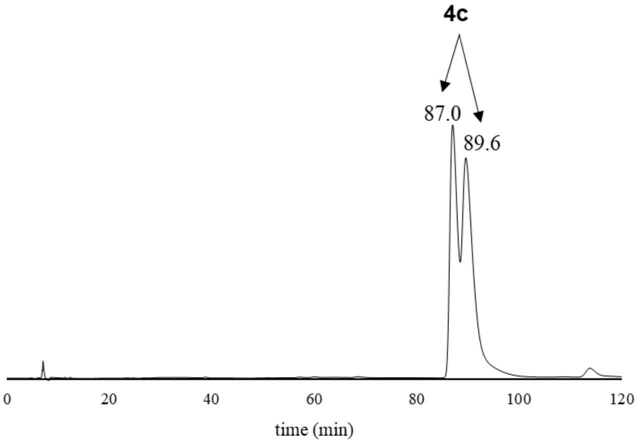

HPLC profile of racemic 5c (Chiralpak IF, eluent: hexane:ethanol = 1:1)
Flow rate = 1.0 mL/min

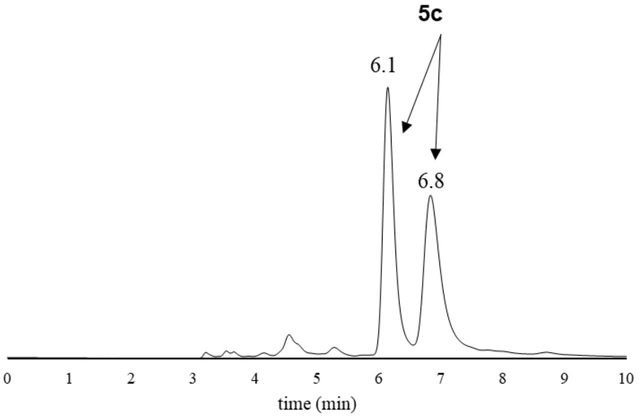

HPLC profile of the dynamic kinetic resolution of racemic 4d to give epoxide 5d
(Chiralpak IF, eluent: hexane:ethanol =1:1)
Flow rate = 1.0 mL/min

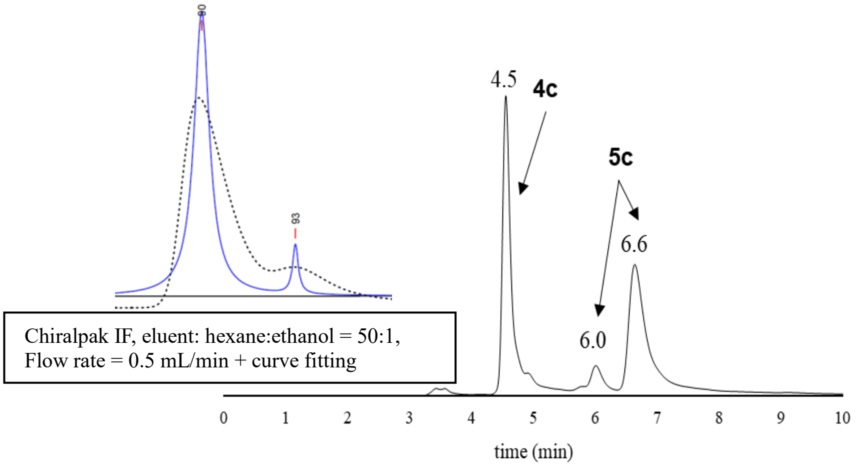

HPLC profile of racemic 4d (Chiralpak IF, hexane)
Flow rate = 0.5 mL/min

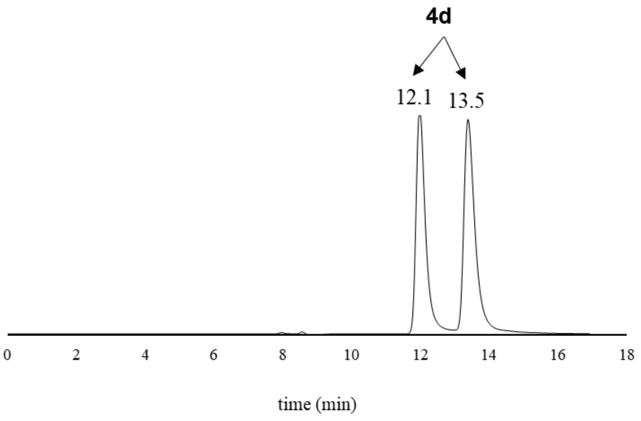

HPLC profile of racemic 5d (Chiralpak IF, eluent: hexane:ethanol = 100:1)
Flow rate = 1.0 mL/min

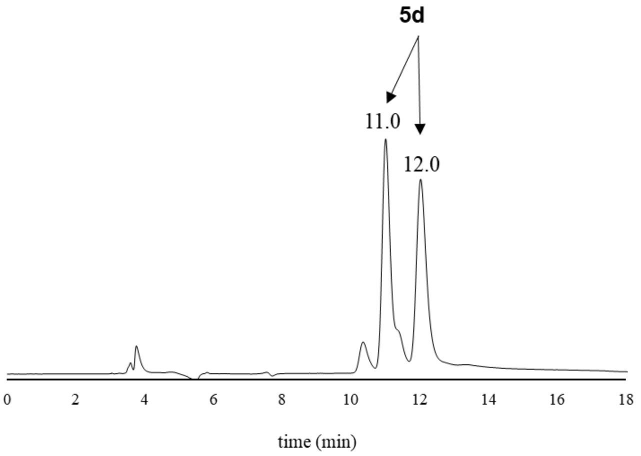

HPLC profile of the kinetic resolution of racemic 4d to give epoxide 5d
(Chiralpak IF, eluent: hexane:ethanol = 100:1)
Flow rate = 1.0 mL/min

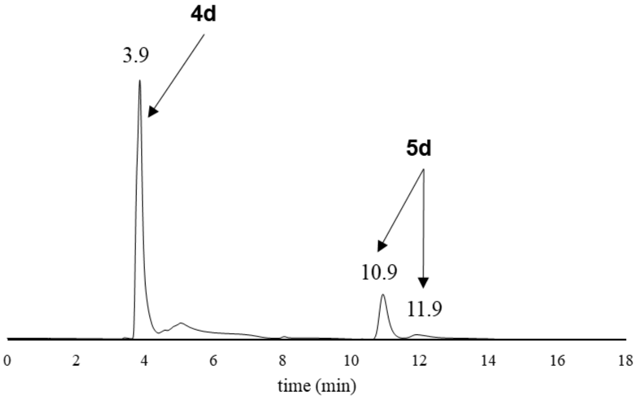

HPLC profile of the kinetic resolution of racemic 4d to give epoxide 5d (unreacted 4d)
Chiralpak IF, eluent: hexane, Flow rate = 0.5 mL/min

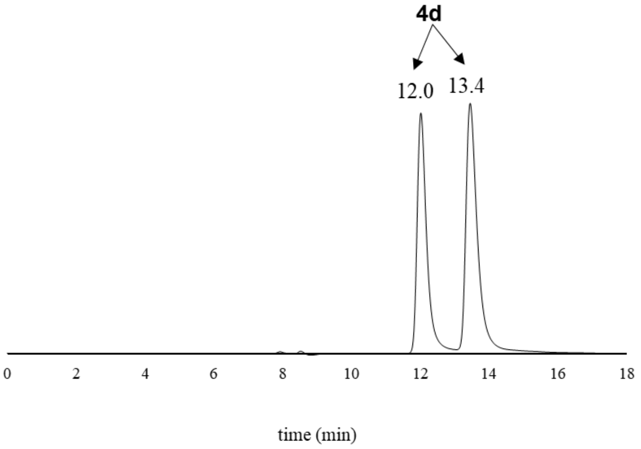

HPLC profile of racemic 4e (Chiralpak IF, eluent: hexane:ethanol = 10:1)
Flow rate = 0.5 mL/min

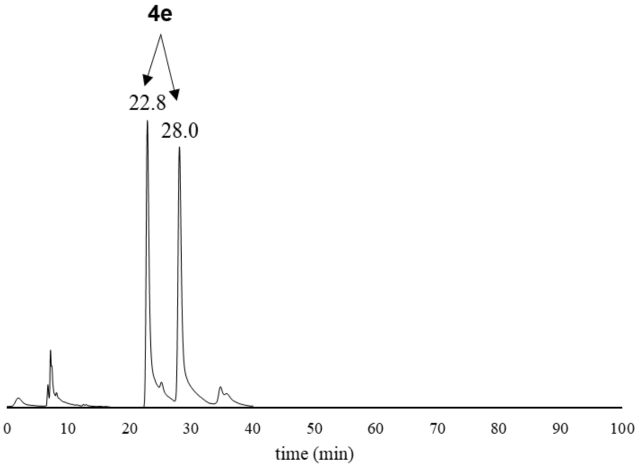

HPLC profile of racemic 5e (Chiralpak IF, eluent: hexane:ethanol =10:1)
Flow rate = 0.5 mL/min

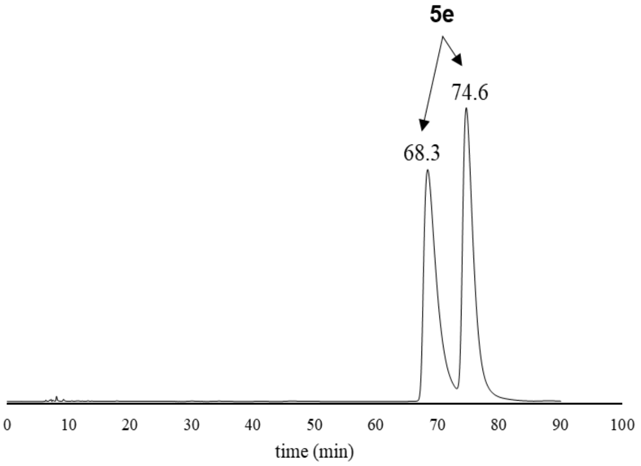

HPLC profile of the dynamic kinetic resolution of racemic 4e to give epoxide 5e (Chiralpak IF, eluent: hexane:ethanol =10:1)
Flow rate = 0.5 mL/min

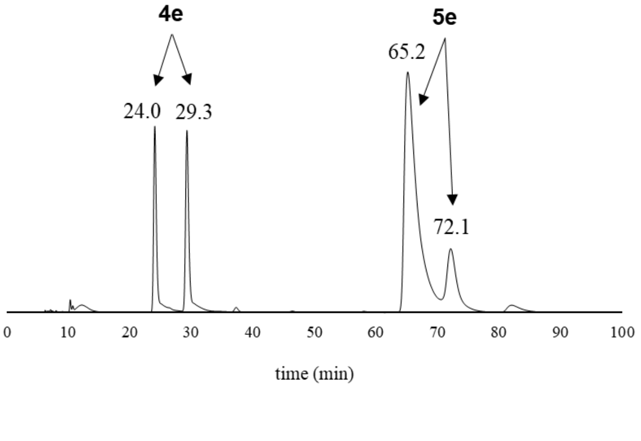

Supplement: Supplementary file 1 — Supplementary Information [file 41598_2018_19878_MOESM1_ESM.pdf]
